# The feasibility of novel point-of-care diagnostics for febrile illnesses at health centres in Southeast Asia: a mixed-methods study

**DOI:** 10.1093/trstmh/trad036

**Published:** 2023-06-15

**Authors:** Fidelis Jacklyn Adella, Moul Vanna, Bipin Adhikari, Sam Ol, Rupam Tripura, Chan Davoeung, James J Callery, Yok Sovann, Arjun Chandna, Voeunrung Bunreth, Carina Asnong, Lorenz von Seidlein, Arjen M Dondorp, Richard J Maude, Yoel Lubell, Bridget Wills, Dysoley Lek, Thomas J Peto

**Affiliations:** Centre for Tropical Medicine and Global Health, Nuffield Department of Medicine, University of Oxford, Oxford, OX3 7LG, UK; Action for Health Development, Battambang 021404, Cambodia; Centre for Tropical Medicine and Global Health, Nuffield Department of Medicine, University of Oxford, Oxford, OX3 7LG, UK; Mahidol Oxford Tropical Medicine Research Unit, Faculty of Tropical Medicine, Mahidol University, Bangkok 10400, Thailand; Action for Health Development, Battambang 021404, Cambodia; Centre for Tropical Medicine and Global Health, Nuffield Department of Medicine, University of Oxford, Oxford, OX3 7LG, UK; Mahidol Oxford Tropical Medicine Research Unit, Faculty of Tropical Medicine, Mahidol University, Bangkok 10400, Thailand; Battambang Provincial Health Department, Battambang, Cambodia; Centre for Tropical Medicine and Global Health, Nuffield Department of Medicine, University of Oxford, Oxford, OX3 7LG, UK; Mahidol Oxford Tropical Medicine Research Unit, Faculty of Tropical Medicine, Mahidol University, Bangkok 10400, Thailand; Pailin Provincial Health Department, Pailin, Cambodia; Centre for Tropical Medicine and Global Health, Nuffield Department of Medicine, University of Oxford, Oxford, OX3 7LG, UK; Cambodia Oxford Medical Research Unit, Angkor Hospital for Children, Siem Reap 171202, Cambodia; Battambang Provincial Health Department, Battambang, Cambodia; Centre for Tropical Medicine and Global Health, Nuffield Department of Medicine, University of Oxford, Oxford, OX3 7LG, UK; Centre for Tropical Medicine and Global Health, Nuffield Department of Medicine, University of Oxford, Oxford, OX3 7LG, UK; Mahidol Oxford Tropical Medicine Research Unit, Faculty of Tropical Medicine, Mahidol University, Bangkok 10400, Thailand; Centre for Tropical Medicine and Global Health, Nuffield Department of Medicine, University of Oxford, Oxford, OX3 7LG, UK; Mahidol Oxford Tropical Medicine Research Unit, Faculty of Tropical Medicine, Mahidol University, Bangkok 10400, Thailand; Centre for Tropical Medicine and Global Health, Nuffield Department of Medicine, University of Oxford, Oxford, OX3 7LG, UK; Mahidol Oxford Tropical Medicine Research Unit, Faculty of Tropical Medicine, Mahidol University, Bangkok 10400, Thailand; Harvard TH Chan School of Public Health, Harvard University, Boston, MA 02115, USA; The Open University, Milton Keynes, MK7 6AA, UK; Centre for Tropical Medicine and Global Health, Nuffield Department of Medicine, University of Oxford, Oxford, OX3 7LG, UK; Mahidol Oxford Tropical Medicine Research Unit, Faculty of Tropical Medicine, Mahidol University, Bangkok 10400, Thailand; Centre for Tropical Medicine and Global Health, Nuffield Department of Medicine, University of Oxford, Oxford, OX3 7LG, UK; Oxford University Clinical Research Unit, Hospital for Tropical Diseases, 764 Vo Van Kiet, Quan 5, Ho Chi Minh City, Vietnam; School of Public Health, National Institute of Public Health, 80, 289 Samdach Penn Nouth St. (289), Phnom Penh, Cambodia; National Centre for Parasitology, Entomology and Malaria Control, 477 Betong, Khan Sen Sok, Phnom Penh, Cambodia; Centre for Tropical Medicine and Global Health, Nuffield Department of Medicine, University of Oxford, Oxford, OX3 7LG, UK; Mahidol Oxford Tropical Medicine Research Unit, Faculty of Tropical Medicine, Mahidol University, Bangkok 10400, Thailand

**Keywords:** feasibility, febrile illnesses, health centres, point-of-care diagnostics

## Abstract

**Background:**

The decline of malaria in Southeast Asia means other causes of fever are increasingly relevant, but often undiagnosed. The objective of this study was to assess the feasibility of point-of-care tests to diagnose acute febrile illnesses in primary care settings.

**Methods:**

A mixed-methods study was conducted at nine rural health centres in western Cambodia. Workshops introduced health workers to the STANDARD(TM) Q Dengue Duo, STANDARD(TM) Q Malaria/CRP Duo and a multiplex biosensor detecting antibodies and/or antigens of eight pathogens. Sixteen structured observation checklists assessed users’ performances and nine focus group discussions explored their opinions.

**Results:**

All three point-of-care tests were performed well under assessment, but sample collection was difficult for the dengue test. Respondents expressed that the diagnostics were useful and could be integrated into routine clinical care, but were not as convenient to perform as standard malaria rapid tests. Health workers recommended that the most valued point-of-care tests would directly inform clinical management (e.g. a decision to refer a patient or to provide/withhold antibiotics).

**Conclusions:**

Deployment of new point-of-care tests to health centres could be feasible and acceptable if they are user-friendly, selected for locally circulating pathogens and are accompanied by disease-specific education and simple management algorithms.

## Introduction

Rapid diagnostic tests (RDTs) can be performed at the point of care and yield immediate results to enable prompt diagnosis. They are typically simple to carry out; some can be administered by non-healthcare professionals or they can be self-administered. RDTs generally have lower sensitivity and specificity compared with reference tests such as PCR, but their affordability and accessibility allow them to be a particularly useful diagnostic tool in settings with less developed health infrastructure.^1,2^

Some RDTs, such as those for malaria and HIV, which are lateral flow immunoassays (LFIAs), have played an instrumental role in reducing morbidity and mortality caused by the respective pathogens. In Cambodia in 2020, 95.5% of all confirmed malaria cases were diagnosed by RDTs,^3^ and 70% of the tests conducted nationwide were performed by community health workers (CHWs).^4^ RDT-based HIV tests are also the backbone of HIV control programmes, reducing the prevalence of HIV 20-fold in Cambodia since the programme scaled up.^5^

The decline in malaria in Southeast Asia means that other causes of fever are of increasing importance, but the aetiologies are largely unknown and sparsely studied. Prominent non-malarial pathogens detected in patients with fever were *Leptospira* spp. (1.4–20.8%), *Orientia tsutsugamushi* (1.4–3.9%), dengue viruses (6.3–17.6%), influenza viruses (2.0–27.2%), hepatitis viruses (22.6%), Japanese encephalitis virus (6.4%) and other bloodborne pathogens.^6–9^ Building upon the successes of RDTs in tackling malaria and HIV-related morbidity and mortality, there is an interest in deploying RDTs for non-malarial febrile illnesses, but few have been deployed and there is limited understanding of their feasibility in primary care settings. Feasibility and acceptability studies can explore the factors that promote or hinder the uptake of these tools in the field. Similar predeployment studies have been conducted in other settings.^10–13^

The primary objective of this study was to explore the perceptions of healthcare workers (HCWs) regarding the feasibility of novel RDT deployment in primary public health facilities (‘health centres’ [HCs]) in western Cambodia. This study was part of a collaborative programme entitled Expanding the roles of village malaria workers: Operational research in Cambodia (CAM-VMW), conducted by the Cambodian Ministry of Health, a local non-governmental organisation (AHEAD) and an academic partner.

## Methods

We conducted a qualitative-dominant mixed-methods study using focus group discussions (FGDs) and structured observation checklists to explore the feasibility of novel RDT deployment in Cambodian HCs. The FGDs were organised after an in-person workshop, in which HCWs were introduced to one of the three RDTs used in this project.

**Figure 1.**
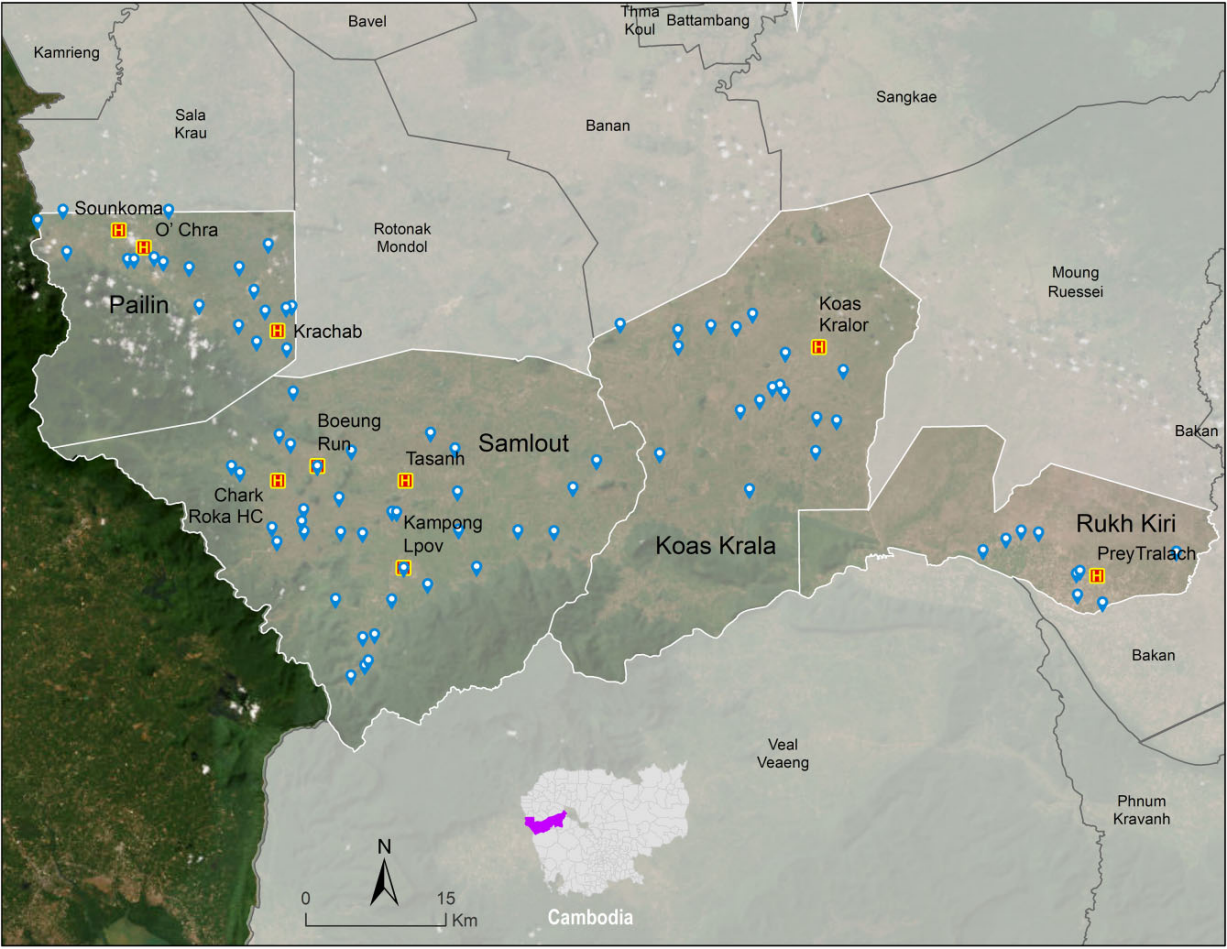
Geographical area in which the project took place, separated into four areas in two provinces: Pailin municipality in Pailin Province, and Samlout, Kosh Kralor and Rukh Kiri districts in Battambang Province. The nine health centres involved in this project are marked ‘H’.

This study was conducted during May–August 2022 in western Cambodia. Participating HCs (Battambang and Pailin provinces, Figure 1) were recruited based on coverage area and study resource constraints. Participating HCs saw a range of 16 to 500 febrile patients per month (fever was the most frequent presentation). Aside from the malaria RDT supplied by the Cambodian National Malaria (CNM) programme, some centres had diagnostic tests for HIV, syphilis, haemoglobin, urinary protein, hepatitis C, blood glucose, TB and pregnancy. None of the HCs had previously used tests for CRP, dengue or a multiplexed RDT. In daily practice, diagnoses were made clinically with support from available tests.

Six to eight participants per HC were chosen to participate, based on convenience. The inclusion criteria were HCWs who work in the HC. The exclusion criteria were unavailability and/or refusal to be included in the study.

### Intervention and data collection

The workshops and FGDs took place in the respective HCs during working hours after most of the patients in the HCs had been attended to, minimising disruption to HC service delivery. The FGDs were conducted immediately after the workshop. The sessions were carried out in Khmer by two facilitators with prior FGD experience.

#### Workshop

The workshop involved a verbal explanation of the test while showing the test device and associated tools. This was followed by a demonstration by the facilitators, and then a session for the participants to practise the test on each other. Printed illustrations were used when necessary to illustrate the possible outcomes of the test. Each HC was introduced to one of the RDT kits below, which would help diagnose diseases suspected to be prevalent in the area^6–9^ (see Table 1 for the accuracy of each test, and Supplementary Data 1 and 2 for detailed practical information on the RDT packages):

1. STANDARD Q Dengue Duo, SD Biosensor There is a high prevalence of dengue cases among febrile patients in Cambodia.^14–16^ The STANDARD Q Dengue Duo detects the non-structural protein-1 (NS1) antigen and pan-serotype immunoglobulin M (IgM) against the dengue virus. The use of combined NS1 and IgM tests has been shown to improve the sensitivity of diagnosis compared with NS1 tests alone.^17^
2. STANDARD Q Malaria/CRP Duo, SD Biosensor The STANDARD Q Malaria/CRP Duo detects histidine-rich protein-2 (HRP-2) specific to *Plasmodium falciparum, Plasmodium* lactate dehydrogenase (pLDH) specific to *Plasmodium* species and CRP, a host biomarker reflecting inflammation at levels >20 mg/L. CRP levels have been shown to discriminate well between bacterial and viral pathogens prevalent in the region.^18^
3. DPP Fever Panel II Asia Antibody and Antigen, Chembio DiagnosticsThe DPP Fever Panels are multiplexed LFIAs that each detect multiple diseases endemic to Southeast Asia. The antigen kit detects Chikungunya, pan-*Plasmodium* pLDH, *P. falciparum* malaria HRP-2, *Burkholderia* Capsular Polysaccharide (CPS, marker of melioidosis), Zika NS1 and Dengue NS1. The antibody kit detects IgM antibodies for Zika, Chikungunya, Dengue, *Rickettsia typhi, O. tsutsugamushi* and *Leptospira* spp. At the time of the study, these kits were not yet commercially available.^19^

**Table 1.** Sensitivity and specificity of the POCTs used in the study

Test component	Cut-off values	Sensitivity	Specificity
**STANDARD(TM) Q Dengue Duo, SD Biosensor** ^a^			
NS1		92.9% (184/198)	98.7% (222/225)
IgM		97.5% (77/79)	96.6% (346/358)
IgG		97.2% (140/144)	96.2% (282/293)
**STANDARD(TM) Q Malaria/CRP Duo, SD Biosensor** ^a^			
P.f.		99.58% (476/478)	100% (1000/1000)
P.v., P.m., P.o.		100% (129/129)	
CRP		87.5% (21/24)	100% (50/50)
**DPP(R) Fever Panel Asia II Antibody and Antigen** ^b^			
*Antigen*			
pLDH malaria	≥19	76% (108/143 malaria cases)	99% (464/471 non-malaria controls)^c^
HRPII malaria	≥9	94% (75/80 P.f. malaria cases)	98% (524/534 non-P.f. malaria controls)^d^
Dengue NS1	≥22	55% (54/98 dengue cases)	95% (491/516 non-dengue controls)^e^
*B. pseudomallei* CPS	≥8	27% (47/177 melioidosis cases)	97% (424/437 non-melioidosis controls)^f^
Chikungunya	≥12	NA	95% (586/614 patients)^g^
Zika NS1	≥11	NA	94% (586/614 patients)^g^
*Antibody*			
Dengue	≥56	11% (11/98 dengue cases)	95% (491/516 non-dengue controls)^e^
Chikungunya	≥21	NA	95% (585/614 patients)^g^
Zika	≥18	NA	95% (585/614 patients)^g^
*Leptospira*	≥36	NA	95% (585/614 patients)^g^
*Orientia tsutsugamushi*	≥7	NA	97% (595/614 patients)^g^
*Rickettsia typhi*	≥10	NA	95% (584/614 patients)^g^
*Used as a combination*			
pLDH + HRPII malaria Ag test	≥19, ≥9	91% (130/143 malaria cases)	97% (458/471 non-malaria controls)
Dengue NS1 Ag + Ab test	≥22, ≥56	61% (60/98 dengue cases)	91% (472/516 non-dengue controls)

The DPP(R) Fever Panel Asia II Antibody and Antigen systems were tested on patients with malaria, dengue, melioidosis and bacteraemia. Patients with bacteraemia included patients with blood culture positive for *Escherichia coli, Klebsiella pneumoniae* and *Staphylococcus aureus*.

a SD Biosensor Product Catalog 2021. SD Biosensor. Available online at https://www.sdbiosensor.com/link/catalogue/2021%20All%20Product%20Catalog_20210601_low.pdf [accessed April 4, 2023].^21^

b Amornchai P, Hantrakun V, Wongsuvan G, et al. Sensitivity and specificity of DPP® Fever Panel II Asia in the diagnosis of malaria, dengue and melioidosis. Journal of Medical Microbiology. 71:001584.^22^

c Included patients with bacteraemia, dengue and melioidosis.

d Included patients with bacteraemia, *Plasmodium vivax* malaria, dengue and melioidosis.

e Included patients with bacteraemia, malaria and melioidosis.

f Included patients with bacteraemia, malaria and dengue.

g Included patients with bacteraemia, malaria, dengue and melioidosis.

#### Structured observation checklists

After the RDT introduction and demonstration, the facilitators assessed the RDT usage of a select group of participants with a structured observation checklist (see Supplementary Data 3). The facilitator graded each step of the procedure with scores of 0 (not done or no comprehension), 1 (done imperfectly or requiring help) or 2 (done perfectly).

#### FGDs

The FGDs were audio-recorded and carried out immediately after the workshop to gain initial impressions from the HCWs and minimise recall bias (FGD guide available as Supplementary Data 4). Short (5–15 min) in-person debriefings with the Cambodian facilitators were carried out at the end of each session.

#### Transcription and translation

The FGD recordings in Khmer were transcribed by an independent local language service provider. The translated transcriptions were reviewed by the research team and FGD facilitators to check for contextual consistency.

#### Data analysis

NVivo version 12 by QSR International, Hawthorn East, VIC, Australia, was used to organise the translated transcripts. Analysis was conducted using a framework analysis.^20^ Emerging codes were derived inductively, then categorised into either ‘enabling factors’, ‘barriers’ or ‘suggestions’. Field notes were consulted to add context during coding.

## Results

### Sociodemographic characteristics of participants

A total of 69 participants were included in the FGDs, 16 of whom were included in the structured observations (see Supplementary Data 5). Twenty-five participants were in the Dengue Duo group; 14 in the Malaria/CRP Duo group; 12 in the DPP Antigen group; and 18 in the DPP Antibody group. The number of FGD participants varied from five to 10 people per HC. Age ranged from 25 to 60 y and 39 participants (55%) were females. The professional background of participants was midwifery, nursing or laboratory technicians. None of the HCWs declined to participate and none withdrew.

### Structured observation checklist

A total of five participants performed the Dengue Duo, three for the Malaria/CRP Duo and four each for the DPP Antigen and DPP Antibody groups. Overall, participants performed well but had trouble during the blood collection for the Dengue Duo (no participant managed to perform the step correctly). It was also notable that participants had difficulties interpreting the antibody arm of the Dengue Duo test (one out of four correct) and using the radiofrequency identification (RFID) reader for the DPP tests. Table 2 depicts the results of the Dengue Duo assessment. Complete results for the other RDTs can be found in Supplementary Data 6.

**Table 2.** Demonstration checklist results for STANDARD(TM) Q Dengue Duo

	Participant				
STANDARD(TM) Q Dengue Duo steps	KK01	PT03	PT02	OC09	OC03
**COMPREHENSION**					
Q0: Dengue knowledge	N/A	N/A	N/A	N/A	N/A
**PROCEDURE**					
Q1: Asepsis and finger prick	2	2	2	2	2
Q2: Collect blood (NS1) with pipette	1	1	1	1	0
Q3: Transfer blood to NS1 device	2	2	2	2	N/A
Q4: Collect blood (AB test) with capillary tube	2	2	2	2	2
Q5: Transfer blood to AB device	2	2	1	2	2
Q6: Add buffer	2	2	2	2	2
**READING AND INTERPRETATION**					
Q7: Read results in 15–20 min	2	2	2	2	2
Q8: NS1 test interpretation	1	N/A	2	2	2
Q9: AB test interpretation	1	N/A	1	2	1

Table 2 gives the assessment results of participants performing the STANDARD(TM) Q Dengue Duo test. Each step of the procedure was graded as 0 (not done), 1 (done imperfectly or requiring help) or 2 (done perfectly). Dengue knowledge was not assessed as the facilitators did not ask the comprehension questions. Step Q3 by participant OC03 could not be assessed because they failed step Q2.

### FGDs

One FGD was conducted in each HC, resulting in a total of nine FGDs. The length of the FGDs ranged from 28 to 51 min.

#### Enabling factors for novel RDT deployment in health centres

##### Perceived usefulness.

All FGD participants expressed that the RDTs provide a more precise diagnosis, were time-saving and supported the appropriate treatment without the need for a laboratory. Participants expected that RDTs could avoid clinical errors that are inevitable during empirical diagnosis, allowing the HCWs to diagnose and treat patients more accurately and efficiently, ultimately guiding the correct prescription of antibiotics, with minimal adverse consequences.

Respondents stated that with increased diagnostic precision, patients would be less inclined to go to private practices and be less frequently referred to faraway hospitals for diagnosis, thus saving money and time. This was regarded as part of the usefulness of an RDT by the participants.

*And for the price, it is expensive if they get it from outside [private] services; and most people do not have much money; most of the patients who come to our health centre everyday use a poor certificate … So, we would like the product which is being introduced to be used with them soon in order to reduce their expense* (Boeung Run HC, Malaria/CRP Duo, FGD with seven participants).

##### Possible integration in workflow.

Participants stated that they would like to use the novel RDTs because they are easy to use and could be integrated into the HC workflow.

*I give a high mark because it is easy to use. We can take it along wherever we go and easily use it one by one. We do not need to use a microscope, and those who have less knowledge about it also can use it* (Chorrk Roka HC, Malaria/CRP Duo, FGD with eight participants).

Participants felt they were able to perform the tests provided they received support, with more practice and/or training. The prior use of RDTs added confidence.

*It is not hard as we have a lot of experience but for the younger people, they have difficulty. Therefore, I still believe that it is good to support and study it more and we must think that while nothing is impossible for us, there are always some difficult points too* (Boeung Run HC, Malaria/CRP Duo).

*We think that it is good and not confusing because it is like the malaria test* (O'Chra HC, Dengue Duo, FGD with nine participants).

To be integrated into the workflow, participants requested that not only should the RDTs be supplied in a timely manner and in sufficient quantity, but HCs should also be supplied with the medications needed to treat the patients should they be diagnosed with an illness based on the RDT results.

##### Patient interest.

HCWs thought that patients sometimes had less confidence in the HCs as they believed the HCs lacked diagnostic capability. HCs mostly use clinical diagnosis, and diagnoses were based on HCWs’ judgement. Novel RDTs would improve patient access to diagnosis, resulting in more patients utilising HC services as their confidence in HCWs’ diagnoses increased.

*They don't come to our side as they have no trust on our health centre due to the fact that the health centre cannot do anything without a test for detection, and then they decide to go to private practice services instead. But if this test is available for detection, they still lack transportation means and a lot of them will come to get the service at our health centre* (Boeung Run HC, Malaria/CRP Duo, FGD with seven participants).

#### Barriers to novel RDT deployment in health centres

##### Technical difficulties.

Technical difficulties seemed to be the main barrier to novel RDT deployment. Data relevant to such technical difficulties are summarised in Supplementary Data 7. HCWs raised concerns that they would need relatively more time and needles for specimen collection if the required amount of blood samples was large, especially when working with paediatric patients. This concern was most pronounced in the Dengue Duo groups.

*As you could see the test just now, it was hard to pump blood for test. So, getting blood for test is not good, and the squeezing of blood is not good either. It causes us to take more time and consumes more needles* (Prey Tralach HC, Dengue Duo, FGD with seven participants).

…*but for the big one [the NS1], if we are careless, it might have air inside the pipette which results in less amount of blood* (O'Chra HC, Dengue Duo, FGD with nine participants).

Some features of the RDT kits were found by the participants to be inconvenient, ranging from the procedures to the multiple sampling tools, buffers and wells. The multiple sample wells were considered to incur errors when depositing the buffer or the specimen, and the multiple sampling tools could cause confusion for an untrained person. Some participants stated that it may be difficult to read the English labels on the buffer bottles and the cassette, as not everyone could read English. The waiting time was a concern for the respondents in the DPP groups, but not by the Dengue Duo and Malaria/CRP Duo groups. This may be because the DPP necessitated an extra waiting time of 5 min (20–25 min in total) compared with the Dengue and Malaria/CRP Duos (15–20 min in total). The DPP required waiting for 5 min before proceeding to the next step of the test, which meant longer active involvement by the HCWs.

*We have three blood sampling tools, one for malaria, but the other two we don't really know. But if we have participated in this training session, we would know that one tube is to collect the blood, and the other tube is for mixing the blood with the buffer and transferring the mix into the well … if we have one test for detection of only one infection then it is not confusing [but in this kit] there are multiple materials with different functions* (Boeung Run HC, Malaria/CRP Duo, FGD with seven participants).

Participants expressed significant concerns regarding the interpretation of quantitative results (DPP RDTs), as they were less intuitive than qualitative results (‘normal’ and ‘abnormal’). With the DPP tests, the unavailability of normal threshold values (cut-offs for determining whether a test was 'positive’) during the training was a barrier for the participants to interpret test results.

*I do not understand the meaning of ‘normal’ or ‘abnormal’. … We have already practised the test, but we only know 2, 3 and 4 (numbers); however, we do not know whether or not there is a disease. Thus, I would like a clearer explanation and request more training courses* (Krachab HC, DPP Antigen, FGD with five participants).

##### Patient response and beliefs in the community.

Participants discussed patients’ dissatisfaction with the relatively larger amount of blood required for the dengue test (110 µl for the dengue test compared with 15 µl for the malaria/CRP test, 50 µl for the DPP Antigen and 10 µl for the DPP Antibody). It was inconvenient having to ask patients to get pricked more than once if the first prick did not give enough blood, as this would cause discomfort for the patients. Furthermore, there was a belief in the community that losing this amount of blood would be harmful to their health.

*Some patients complain about us getting more blood. For a real example on social media, someone said his mother's blood pressure went up to 150 [mmHg, SBP], and by taking four syringes of his mother's blood, her blood pressure was lowered to 100 [mmHg]. Now his mother got a bit better, he really thought that way. So, he was not very happy with us getting more blood* (O'Chra HC, Dengue Duo, FGD with nine participants).

Participants thought that patients who received a positive RDT result and got referred to higher levels of health facilities would undergo repeat testing at each referral point. They were worried that patients would not like to be tested multiple times.

#### HCWs’ suggestions or ideas for the deployment of novel RDTs

##### Improvements to the RDTs.

The HCWs recommended that if an RDT package was deployed, it should contain everything needed to perform the test, including lancets and alcohol swabs in sufficient quantity, a tube stand and a timer if needed. Participants suggested that every individual test device supplied by the manufacturer should contain all the necessary materials required to perform the test, such as one test device with several drops of buffer, several lancets and one inverted cup to ease the conducting of these tests away from the HC.

Respondents suggested some physical features of the tests be improved to avoid mix-ups, such as labelling similar materials and tools (e.g. buffers, wells) with distinct colours or numbers. Participants preferred to have the labels written in the local language. Interpretation of the results could be made more intuitive. For the DPP multiplex, the participants preferred to have results displayed as normal/abnormal, or with a distinctive feature to indicate a ‘positive result’.

##### Manual documents and guidelines.

Participants from all RDT FGDs suggested that simple printed instructions detailing how to conduct the tests and interpret the results would be needed (instruction sheets in English were provided by the manufacturers but were not circulated in the workshops because most of the participants did not understand English and also due to time limitations). Furthermore, any novel RDTs would need to be authorised by the Ministry of Health and included in the national health programme prior to deployment.

*We do not have papers to read, so we might forget it. So, we also want a manual for reading when we forget it* (Prey Tralach HC, Dengue Duo, FGD with seven participants).

*The result is not clear what level is normal and what level is abnormal; what level is negative … I would like a proper guideline for convenience* (Soun Koma HC, DPP Antigen, FGD with seven participants).

##### Qualifications and training.

If RDTs were to be deployed, participants generally agreed that more/all HC staff should be trained in the procedures and that the training should be more extensive than that provided during this project. The training should provide instructions on how to conduct the tests as well as background knowledge of the diseases that they would be testing for. Having the opportunity to practise was seen as particularly important for the Dengue Duo, which required a comparatively large blood volume.

If the novel RDTs were to be deployed outside of the HC setting, then everyone expected to carry out the tests should receive sufficient training. Volunteers (CHWs) would be able to carry out the Dengue Duo and Malaria/CRP Duo, but they would need more training sessions compared with HCWs. We did not ask the HCWs whether CHWs would be able to carry out the DPP RDTs because it was unlikely that DPP RDTs would be deployed at the community level due to their complexity.

*Q: Regarding the village volunteers, do you have any ideas to help them use this test properly? A: We should allow them to participate in the training with us; and there should be a bit more training for them together with physicians because they have limited understanding and knowledge. So, it is good if we could have many trainings for them before applying the real work* (Prey Tralach HC, Dengue Duo, FGD with seven participants).

*Because all of us are midwives and working in the nursery we do not have deep understanding, so if possible we would like more training because the staff at my centre have multiple work, meaning they can work in every section other than midwifery work. So we would like more training so we will have deep understanding and convenience when using it* (Krachab HC, DPP Antigen, FGD with five participants).

## Discussion

This study, to the best of our knowledge, is the first exploration of the feasibility of deploying several novel RDTs in Cambodian HCs. Novel RDTs could improve the diagnosis and treatment available at a healthcare facility that is accessible to the patient. Additional requirements must be met for these novel RDTs to be fully utilised. Our findings largely align with earlier studies exploring the feasibility and acceptability of deploying point-of-care tests (POCTs) in field settings.^10–13,23–25^

The deployment of new RDTs in Cambodian HCs could be well accepted as HCWs expressed views that these could be useful and integrated into existing workflows. Participants were motivated to have the RDTs in their HCs and believed that they could improve patient care, as fever was a significant presenting symptom in the HCs, and may be caused by communicable diseases.^26^ Similar results were found by other studies; HCWs perceived providing better quality of care when an RDT was provided to the facility, and conversely burdened with feelings of guilt and shame when failing to provide adequate care.^10–13,23,25^ Young et al. described that HCWs believed that RDTs would improve confidence in diagnosis due to their observability, compared with just relying on clinical diagnosis.^12^ The perception that the new RDTs would improve patient care and the HCWs’ motivation may be linked, as a sense of meaning and autonomy are both associated with job satisfaction in healthcare services.^27,28^

Barriers to novel deployment revealed during this project were predominantly technical. As seen with the RDTs used in this project, the participants had trouble with large-quantity sample collection (specifically for the Dengue Duo), the testing procedure (concern about mix-ups and trouble operating the RFID reader) and the interpretation of results (understanding the concept of antigens and antibodies, and interpreting quantitative results). Complex test procedures and difficult sample collection have been described as barriers to RDT implementation in other studies.^10,13,23^ Technical difficulties were also found to be a bottleneck in deploying novel quantitative POC in Cambodia.^25^

Despite the challenges seen in this project, RDT use in primary care and at community level largely proved feasible, as was observed earlier with RDTs for malaria and HIV, as well as prototype POCTs.^29,30^ The combined dengue RDT has been successfully used in Malaysian primary care, with a note that the accuracy of this test and its impact on clinical management was technician skill-dependent.^31,32^ Our results also indicated buy-in from the HCWs for novel RDT deployment. This suggests that deployment may be feasible if accompanied by adequate training and guidelines, and that intuitive test kits are pivotal to the success of deployment.

The results of our study corroborated the need for ongoing training.^10,11,13,24,25^ Procedures initially found to be difficult can be accomplished with more training and practice.^10,23^ Training should include start-to-finish instructions, minimise abstract concepts (e.g. including thresholds for the DPP RDT interpretation) and enhance participant comprehension. As different levels of instruction or training are needed for different devices in different settings, the appropriate level of training for each provider must be established before deployment.^33^ The manual or guideline, which was requested by the HCWs, should be provided ideally in the local language, as some respondents stated that some of the HCWs could not read and understand English. POCTs such as RDTs should be made as intuitive as possible to maximise accuracy and workflow efficiency.^13,34^ Result thresholds for the DPP multiplex, for example, should be settled on and the reader programmed to provide qualitative results. Furthermore, the availability of POCTs and the consistency of the supply chain for both the RDTs and the therapeutic options must be ensured to make real changes in community health.^10–12^ Dissemination workshops will be held involving local stakeholders and policymakers and the results of this study and the workshop will be shared with the diagnostic manufacturers. Future directions may include operational research or trials to deploy these or similar diagnostics in routine treatment practice to evaluate their impact on case management of febrile illness.

The strength of this study is that it was designed in collaboration with a local NGO (AHEAD) and the national malaria control programme (i.e. CNM), so that pragmatic decisions were tailored to the local context. However, necessary compromises resulted in some limitations. Data saturation for the FGDs and the minimum sample size for the structured observation checklists could not be reached due to time limitations. For a similar reason, printed instructions in the local language on how to use the tests were not prepared and used in the workshop. Participants’ comprehension and skill may have been better if written instructions were provided. Because of financial and human resource constraints, participant identification and recruitment were conducted by convenience sampling, and the sites included were limited to nine HCs. The authors who conducted the data cleaning and analysis did not speak the local language of Khmer (in which the data collection was performed), possibly introducing biases. To mitigate this, field notes obtained from observations and discussions with the AHEAD team were held during data processing.

In conclusion, novel RDTs can be pivotal in improving diagnosis and treatment at the first point of contact. However, we found difficulties for the specific RDTs used in this study, the most prominent being sample collection for the larger quantity of blood (Dengue Duo), the complexity of the test procedure (Malaria/CRP Duo) and the quantitative result interpretation (DPP Fever Panel Asia II). Based on this study, the feasibility of rolling out RDTs could be improved by (1) enhancing the user-friendliness of novel RDTs (e.g. designing technically less demanding kits; designing easily interpretable RDTs, providing guidelines on use and interpretations of the test results in the local language); and (2) providing adequate training and support in using these novel RDTs. Findings from this study can inform the future of POC diagnostics.

## Supplementary Material

trad036_Supplemental_Files

## Data Availability

Data collected for the study, including deidentified individual participant data and a data dictionary defining each field in the set, will be made available to others. These data will be available with publication via the MORU data sharing committee, Chairperson, Professor Phaikyeong Cheah, phaikyeong@tropmedres.ac. Access will be provided for analysis by bona fide researchers with or without investigator support, after approval of a proposal, and upon a signed data access agreement.

## References

[1] Boyce MR , O'Meara WP. Use of malaria RDTs in various health contexts across sub-Saharan Africa: a systematic review. BMC Public Health. 2017;17:470. 28521798 10.1186/s12889-017-4398-1 PMC5437623

[2] Mpina M , Stabler TC, Schindler T et al. Diagnostic performance and comparison of ultrasensitive and conventional rapid diagnostic test, thick blood smear and quantitative PCR for detection of low-density Plasmodium falciparum infections during a controlled human malaria infection study in Equatorial Guinea. Malar J. 2022;21:99. 35331251 10.1186/s12936-022-04103-y PMC8943516

[3] World Health Organization . World Malaria Report 2021. World Health Organization, 2021.

[4] Cambodia Malaria Elimination Action Framework 2021-2025. Phnom Penh: National Center for Parasitology, Entomology and Malaria Control, 2021.

[5] Vun MC , Fujita M, Rathavy T et al. Achieving universal access and moving towards elimination of new HIV infections in Cambodia. Journal of the International AIDS Society. 2014;17:18905. 24950749 10.7448/IAS.17.1.18905 PMC4065309

[6] Mueller TC , Siv S, Khim N et al. Acute undifferentiated febrile illness in rural Cambodia: a 3-year prospective observational study. PLoS One. 2014;9:e95868. 24755844 10.1371/journal.pone.0095868 PMC3995936

[7] Kasper MR , Blair PJ, Touch S et al. Infectious etiologies of acute febrile illness among patients seeking health care in south-central Cambodia. Am J Trop Med Hyg. 2012;86:246–53. 22302857 10.4269/ajtmh.2012.11-0409 PMC3269275

[8] Chheng K , Carter MJ, Emary K et al. A prospective study of the causes of febrile illness requiring hospitalization in children in Cambodia. PLoS One. 2013;8:e60634. 23593267 10.1371/journal.pone.0060634 PMC3621876

[9] Blair PJ , Wierzba TF, Touch S et al. Influenza epidemiology and characterization of influenza viruses in patients seeking treatment for acute fever in Cambodia. Epidemiol Infect. 2010;138:199–209. 19698213 10.1017/S095026880999063X

[10] Asiimwe C , Kyabayinze DJ, Kyalisiima Z et al. Early experiences on the feasibility, acceptability, and use of malaria rapid diagnostic tests at peripheral health centres in Uganda-insights into some barriers and facilitators. Implement Sci. 2012;7:5. 22269037 10.1186/1748-5908-7-5 PMC3398266

[11] Mohamed Y , Kupul M, Gare J et al. Feasibility and acceptability of implementing early infant diagnosis of HIV in Papua New Guinea at the point of care: a qualitative exploration of health worker and key informant perspectives. BMJ Open. 2020;10:e043679. 10.1136/bmjopen-2020-043679 PMC7678362 33444219

[12] Young N , Achieng F, Desai M et al. Integrated point-of-care testing (POCT) for HIV, syphilis, malaria and anaemia at antenatal facilities in western Kenya: a qualitative study exploring end-users’ perspectives of appropriateness, acceptability and feasibility. BMC Health Serv Res. 2019;19:74. 30691447 10.1186/s12913-018-3844-9 PMC6348645

[13] Zongo S , Carabali M, Munoz M et al. Dengue rapid diagnostic tests: health professionals’ practices and challenges in Burkina Faso. SAGE Open Med. 2018;6:2050312118794589. 30147936 10.1177/2050312118794589 PMC6100125

[14] Vong S , Khieu V, Glass O et al. Dengue incidence in urban and rural Cambodia: results from population-based active fever surveillance, 2006-2008. PLoS Negl Trop Dis. 2010;4:e903. 21152061 10.1371/journal.pntd.0000903 PMC2994922

[15] Wichmann O , Yoon I-K, Vong S et al. Dengue in Thailand and Cambodia: an assessment of the degree of underrecognized disease burden based on reported cases. PLoS Negl Trop Dis. 2011;5:e996. 21468308 10.1371/journal.pntd.0000996 PMC3066139

[16] Vong S , Goyet S, Ly S et al. Under-recognition and reporting of dengue in Cambodia: a capture-recapture analysis of the National Dengue Surveillance System. Epidemiol Infect. 2012;140:491–9. 21733251 10.1017/S0950268811001191

[17] Chong ZL , Sekaran SD, Soe HJ et al. Diagnostic accuracy and utility of three dengue diagnostic tests for the diagnosis of acute dengue infection in Malaysia. BMC Infect Dis. 2020;20:210. 32164538 10.1186/s12879-020-4911-5 PMC7069157

[18] Lubell Y , Blacksell SD, Dunachie S et al. Performance of C-reactive protein and procalcitonin to distinguish viral from bacterial and malarial causes of fever in Southeast Asia. BMC Infect Dis. 2015;15:511. 26558692 10.1186/s12879-015-1272-6 PMC4642613

[19] Chembio and FIND collaborate to develop point-of-care multiplex test for acute febrile illnesses in Asia Pacific. FIND. Available at: https://www.finddx.org/newsroom/chembio-and-find-collaborate-to-develop-point-of-care-multplex-test-for-acute-febrile-illnesses-in-asia-pacific/ [accessed June 29, 2022].

[20] Ritchie J , Lewis J, Nicholls CM et al. Qualitative Research Practice: A Guide for Social Science Students and Researchers. 2nd ed. London, UK: SAGE, 2013.

[21] SD Biosensor Product Catalog 2021. SD Biosensor. Available at: https://www.sdbiosensor.com/link/catalogue/2021%20All%20Product%20Catalog_20210601_low.pdf [accessed April 4, 2023].

[22] Amornchai P , Hantrakun V, Wongsuvan G et al. Sensitivity and specificity of DPP® Fever Panel II Asia in the diagnosis of malaria, dengue and melioidosis. J Med Microbiol. 2022;71:001584. 10.1099/jmm.0.001584 PMC7613707 35994523

[23] Bancone G , Gilder ME, Win E et al. Technical evaluation and usability of a quantitative G6PD POC test in cord blood: a mixed-methods study in a low-resource setting. BMJ Open. 2022;12:e066529. 10.1136/bmjopen-2022-066529 PMC9748916 36523222

[24] Zhang Y , Guy R, Camara H et al. Barriers and facilitators to HIV and syphilis rapid diagnostic testing in antenatal care settings in low-income and middle-income countries: a systematic review. BMJ Global Health. 2022;7:e009408. 10.1136/bmjgh-2022-009408 PMC9628546 36319030

[25] Adhikari B , Tripura R, Dysoley L et al. Glucose 6 Phosphate Dehydrogenase (G6PD) quantitation using biosensors at the point of first contact: a mixed method study in Cambodia. Malar J. 2022;21:282. 36195916 10.1186/s12936-022-04300-9 PMC9531219

[26] Global Health Estimates 2020 *:* Deaths by Cause, Age, Sex, by Country and by Region, 2000-2019. Geneva, Switzerland: World Health Organization, 2020.

[27] Clari M , Gonella S, Gatti P et al. Multi-level analysis of individual and work environment factors associated with nurses’ perceived emotional exhaustion. Appl Nurs Res. 2022;63:151514. 35034707 10.1016/j.apnr.2021.151514

[28] Han RM , Carter P, Champion JD. Relationships among factors affecting advanced practice registered nurses’ job satisfaction and intent to leave: a systematic review. J Am Assoc Nurse Pract. 2018;30:101–13. 29757821 10.1097/JXX.0000000000000006

[29] Figueroa C , Johnson C, Ford N et al. Reliability of HIV rapid diagnostic tests for self-testing compared with testing by health-care workers: a systematic review and meta-analysis. Lancet HIV. 2018;5:e277–90. 29703707 10.1016/S2352-3018(18)30044-4 PMC5986793

[30] Paz-Soldan VA , Morrison AC, Sopheab H et al. Potential use of community-based rapid diagnostic tests for febrile illnesses: formative research in Peru and Cambodia. PLoS NeglTrop Dis. 2019;13:e0007773. 10.1371/journal.pntd.0007773 PMC6837536 31658252

[31] CPG Management of Dengue Infection In Adults. 3rd ed. Putrajaya: Ministry of Health Malaysia, 2015.

[32] Andries A-C , Duong V, Ngan C et al. Field evaluation and impact on clinical management of a rapid diagnostic kit that detects dengue NS1, IgM and IgG. PLoS Negl Trop Dis. 2012;6:e1993. 23301110 10.1371/journal.pntd.0001993 PMC3531494

[33] Morrison AC , Schwarz J, Mckenney JL et al. Potential for community based surveillance of febrile diseases: feasibility of self-administered rapid diagnostic tests in Iquitos, Peru and Phnom Penh, Cambodia. PLoS Negl Trop Dis. 2021;15:e0009307. 33901172 10.1371/journal.pntd.0009307 PMC8101991

[34] Chin CD , Linder V, Sia SK. Commercialization of microfluidic point-of-care diagnostic devices. Lab Chip. 2012;12:2118–34. 22344520 10.1039/c2lc21204h

