# Supplementary material for: The feasibility of novel point-of-care diagnostics for febrile illnesses at health centres in Southeast Asia: a mixed-methods study
Source: Trans R Soc Trop Med Hyg. 2023 Jun 15;117(11):788–96. doi: 10.1093/trstmh/trad036 (PMC10629948; doi:10.1093/trstmh/trad036)
Supplement: trad036_Supplemental_Files [file trad036_supplemental_files.zip › Supplementary data 1.pdf]

Malaria/CRP checklist

No	Description	Score			Assessor's notes (please note the details or circumstances of the error)
		0	1	2	
Comprehension					
1	Ask the participants a few simple questions<br>- Malaria<br>o What is malaria caused by? *(malaria is caused by a parasite and spread through mosquitos)*<br>o What are the consequences of malaria? *(fever and sometimes more serious illness/severe malaria)*<br>- CRP<br>o What is CRP? *(CRP is a protein that the body makes in response to infection)*<br>o What does a high CRP level indicate? *(a bacterial infection is likely)*				
Malaria test					
1	Wipe patient's finger with an alcohol wipe and prick with a lancet.				
2	Collect the blood with the blood sample device (5µl)				
3	Transfer the blood to the specimen well (circle well) of the test device				
4	Add 3 drops of buffer to the square well of the test device				
CRP test					
5	Collect the blood with the Ezi tube, filling it to the black line (10µl)				
6	Add the blood to the assay diluent. Dispose the Ezi tube.				
7	Use the dropper (100µl) to mix the specimen by pressing and releasing the bulb 6-8 times.				
8	Collect all specimen using the dropper and add all to the sample well.				
Reading the results					
9	Read both results at 15-20 minutes. Do not read after 20 minutes.				
10	Reading the results: malaria test (use pictures to test participants' knowledge in test interpretation)<br>- The C line must always be visible. Otherwise the test is invalid<br>- Line on "PAN": Pm / Pv / Po positive or a mix of these<br>- Line on "Pf": Pf positive<br>- Lines on both "PAN" and "Pf": Pf positive or mixed Pf with Pv/Po/Pm<br>- No lines on both "PAN" and "Pf": malaria negative				
11	Reading the results: CRP (use pictures to test participants' knowledge in test interpretation)<br>- The C line must always be visible. Otherwise the test is invalid<br>- Line on "T": CRP > 20mg/L (positive)<br>- No line on "T": CRP < 20mg/L (negative)				

Scoring
- 0 : not done
- 1 : done, but incorrectly
- 2 : done correctly

Dengue Duo checklist

No	Description	Score			Assessor's notes (please note the details or circumstances of the error)
		0	1	2	
Comprehension					
1	Ask the participants a few simple questions<br>- Dengue<br>o What is dengue caused by? *(dengue is caused by a virus and spread by mosquitos)*<br>o What are the consequences of dengue? *(fever/febrile illness. Sometimes can be severe: dengue hemorrhagic shock or dengue shock syndrome)*				
NS1 Test					
1	Wipe patient's finger with an alcohol wipe and prick with a lancet.				
2	Collect the blood using the sample device, filling it to the black line (100µl)				
3	Transfer the blood to the NS1 test device.				
Antibody test					
4	Collect the blood using the Ezi tube, filling it to the black line (10µl)				
5	Transfer the blood to the specimen well (smaller well) of the test device				
6	Add 3 drops of buffer to the square well of the test device				
Reading the results					
7	Read both results at 15-20 minutes. Do not read after 20 minutes.				
8	Reading the results: NS1 (use pictures to test participants' knowledge in test interpretation)<br>- The C line must always be visible. Otherwise the test is invalid<br>- Line on "T": positive for dengue<br>- Negative on "T": negative for dengue				
9	Reading the results: dengue antibody (use pictures to test participants' knowledge in test interpretation)<br>- The C line must always be visible. Otherwise the test is invalid<br>- Line on "M": positive for dengue IgM<br>- Line on "G": positive for dengue IgG<br>- Lines on both "M" and "G": positive for dengue IgM and IgG<br>- No lines on both "M" and "G": dengue negative				

<u>Scoring</u>
- 0 : not done
- 1 : done, but incorrectly
- 2 : done correctly

DPP Febrile Panel Checklist (Antigen)

No	Description	Score			Assessor's notes (please note the details or circumstances of the error)
		0	1	2	
DPP Febrile Panel Antigen Test					
1	Wipe patient's finger with an alcohol wipe and prick with a lancet.				
2	Collect the blood with the 50µl capillary tube by touching the blood horizontally, filling it to the black line.				
3	Transfer the blood to the "Sample+Buffer Well 1" of the test device.				
4	Add 4 drops of the Sample Buffer to the same well ("Sample+Buffer Well 1"				
5	Start a timer				
6	At 5 minutes, add 12 drops of the Running Buffer to the "Buffer Well 2"				
Reading the results					
7	At 20-25 minutes, read the results with the DPP Micro Reader 2.				
8	Set up the DPP Micro Reader 2 to read the antigen test using the Antigen RFID card.				
9	Put in the test device into the reader slot and read the results.				

<u>Scoring</u>
- 0 : not done
- 1 : done, but incorrectly
- 2 : done correctly

DPP Febrile Panel Checklist (IgM Antibody)

No	Description	Score			Assessor's notes (please note the details or circumstances of the error)
		0	1	2	
DPP Febrile Panel IgM Antibody Test					
1	Add 5 drops of the Sample Buffer into the sample vial.				
2	Wipe patient's finger with an alcohol wipe and prick with a lancet.				
3	Collect the blood with the 10µl capillary tube by touching the blood horizontally, filling it to the black line.				
3	Transfer the blood to the sample vial. Discard the capillary tube.				
4	Using the 100µl transfer pipette, mix the blood and the buffer by pipetting up and down 6-8 times. Do not introduce air bubbles to the solution.				
5	Using the same transfer pipette, collect the specimen by filling it up to the black line.				
6	Release all the solution into the "Sample+Buffer Well 1".				
7	Start a timer				
8	At 5 minutes, add 12 drops of the Running Buffer to the "Buffer Well 2"				
Reading the results					
9	At 20-25 minutes, read the results with the DPP Micro Reader 2.				
10	Set up the DPP Micro Reader 2 to read the IgM test using the IgM RFID card.				
11	Put in the test device into the reader slot and read the results.				

Scoring
- 0 : not done
- 1 : done, but incorrectly
- 2 : done correctly
