# Supplementary material for: The feasibility of novel point-of-care diagnostics for febrile illnesses at health centres in Southeast Asia: a mixed-methods study
Source: Trans R Soc Trop Med Hyg. 2023 Jun 15;117(11):788–96. doi: 10.1093/trstmh/trad036 (PMC10629948; doi:10.1093/trstmh/trad036)
Supplement: trad036_Supplemental_Files [file trad036_supplemental_files.zip › Supplementary data 2.pdf]

គោល ការណ៍ ណែ នាំ ពិភាក្សា ផ្តោត លើ ក្រុមបុគ្គលិក មណ្ឌល សុខ ភាព ៖
ការ វាយ តម្លៃ ដោយ ផ្អែក លើ មណ្ឌល សុខភាព នៃ ការធ្វើរោគ វិនិច្ឆ័យ ថ្មី
Focus group discussion guidelines for health centre staff:
Health centre-based evaluation of novel diagnostics

ឈ្មោះ គម្រោង ៖ ការ ពង្រីក តួនាទី ភ្នាក់ងារគ្រុនចាញ់ ភូមិ ៖ តាមការ ស្រាវជ្រាវ ប្រតិបត្តិការ នៅ កម្ពុជា

Project name: Expanding the roles of village malaria workers: Operational research in Cambodia

ចំណាំ: ការប្រើប្រាស់មគ្គុទ្ទេសក៍នេះត្រូវបានគេរំពឹងថានឹងអាចបត់បែនបាន ហើយនឹងត្រូវបានកែសម្រួលសម្រាប់ FGDs និង SSIs ។ មគ្គុទ្ទេសក៍ នេះ ត្រូវ បាន រំពឹង ថា នឹង ជួយ អ្នក សម្ភាសន៍ ឲ្យ នៅ ក្នុង កំហិត នៃ ប្រធាន បទ ដែល ដឹក នាំ ដោយ សំណួរ ស្រាវជ្រាវ នេះ ។ សំណួរ ដែល បាន បញ្ចូល ខាង ក្រោម នេះ គឺ ជា ឧទាហរណ៍ តែ ប៉ុណ្ណោះ ហើយ គួរ សម្រប ខ្លួន ក្នុង អំឡុង ពេល ពិភាក្សា/សម្ភាស។ ការ អង្កេត និង សំណួរ ដែល មាន សក្តានុពល នៅ ក្រោម ប្រធាន បទ អាច ត្រូវ បាន ឃ្លាំមើល ឡើង វិញ និង ត្រូវ បាន សួរ តាម របៀប ដែល អ្នក ជា អ្នក សម្ភាសន៍ គិត ថា សមរម្យ ។ អ្នក អាច បន្ថែម សំណួរ ដើម្បី ប្រមូល ព័ត៍មានសំខាន់ៗ បន្ថែម

Note: Use of this guide is expected to be flexible and will be adapted for FGDs and SSIs. The guide is expected to help interviewer stay within the confines of the themes guided by the research question. Questions included below are only examples and should be adapted during the discussions/interviews. Probes and potential questions under the themes can be re-phrased and asked in ways you as an interviewer think appropriate. You may add questions/probes to collect additional and important information.

១. សំនួរណែនាំ និង សាវតា

1. Introduction and background questions

អរគុណច្រើនចំពោះការចូលរួមក្នុងកិច្ចពិភាក្សានេះ។ កិច្ចពិភាក្សានេះនឹងចំណាយពេល ៦០-៩០នាទី។ យើង នឹង ពិភាក្សា សំណួរ ទាក់ទង នឹង បទពិសោធន៍ របស់ អ្នក ជាមួយ ឧបករណ៍ ដែល យើង បាន ប្រើ នៅ ក្នុង សិក្ខាសាលា ។ ការ ពិភាក្សា នេះ នឹង ត្រូវ បាន កត់ ត្រាថតចម្លង ដោយ អូឌីយ៉ូ ដើម្បី អនុញ្ញាត ឲ្យ មាន ឯកសារ ត្រឹម ត្រូវ នៃ ការ ពិភាក្សា ។ នៅ ពេល ណា ក៍ ត(ត្រូវតែបញ្ជាក់ឲ្យបានច្បាស់ពីគោលបំណងនៃការពិភាក្សានេះ)ដោយបើ អ្នក មាន អារម្មណ៍ ថា យើង ចង់ ឈប់ ឬ សម្រាក សូម ប្រាប់ ខ្ញុំ។

Thank you very much for participating in this discussion. The discussion will take 60-90 minutes. We will be discussing questions regarding your experience with the kit we used in the workshop. This discussion will be audio-recorded to enable accurate documentation of the discussion. If at any time you feel that we need to stop or take a break, please let me know.

ដើម្បីចាប់ផ្តើម តើអ្នកអាចប្រាប់ខ្ញុំពីប្រវត្តិនិងការងារបច្ចុប្បន្នរបស់អ្នកបានទេ?

To begin with, could you tell me something about your background and current work?

ព័ត៌មានទូទៅ
General Information

Participant number:

លក្ខណៈ ប្រជា សាស្ត្រ សង្គម របស់ អ្នក ឆ្លើយដែលត្រូវឆ្លើយ តប<br>Sociodemographic characteristics of respondents	១-អាយុ<br>1. Age	
	២-ភេទ<br>2. Gender	ប្រុសMale □ ស្រីFemale □ ផ្សេងៗ Other □
	៣ ការងារ<br>3. Occupation	
	៤. ការងារ<br>4. Workplace	e.g., xx មណ្ឌលសុខភាព health centre.
	៥. ឈ្មោះ ភូមិ<br>5. Village Name	
	៦. កំរិតវប្បធម៌/ អប់រំ<br>6. Qualification/ Education	

<table>
<tr><th colspan="2">ការ វិនិច្ឆ័យ ថ្មី សម្រាប់ គ្រប់គ្រង ជំងឺ គ្រពះ និង ការ ប្រើប្រាស់ របស់ វា

Novel diagnostics for managing febrile illness and their use</th></tr>
<tr><th>Themes</th><th>មគ្គុទ្ទេស Guide</th></tr>
<tr><td>លក្ខណៈ នៃ ការងារ របស់ បុគ្គលិក សុខភាព មុន ពេល ទទួល ភារកិច្ច ធ្វើការវិនិច្ឆ័យនិងផ្តល់រោគវិនិច្ឆ័យដោយប្រើតេស្តNovel RDT
Characteristics of health workers’ work before tasked with novel diagnostics</td><td>1. តើ អ្នក កំពុងបំពេញ ការងារ របស់ អ្នក យ៉ាង ដូចម្តេចខ្លះ ?ធ្វើអ្វីខ្លះ?
How are you doing with your job?
2. តើ អ្នក បានជួប អ្នក ជំងឺ គ្រុន ក្តៅ ប៉ុន្មាន នាក់ ក្នុង មួយ ខែ ?
How many fever patients do you see in a month?
3. តើអ្នកដឹងថាអ្នកជំងឺគ្រុនក្តៅទាំងនេះបណ្តាលពីអ្វីខ្លះ?
What are these fever patients suffering from?
4. តើ ពួក គេ មាន ជំងឺ គ្រុន ឈាម ឬ ជម្ងឺបណ្តាលមកពីមេរោគវារ៉ុស ផ្សេង ទៀត ដែរ ឬ ទេ ?
(សូមបញ្ជាក់អពីជម្ងឺដែលបណ្តាលមកពីគ្រុនក្តៅ)
Do they have dengue, viral infections any other febrile illnesses? (use prompts to list out febrile illnesses)
5. តើ ការធ្វើរោគវិនិច្ឆ័យចំពោះអ្នកមានគ្រុនក្តៅដោយផ្អែកលើអ្វីដែរ?ពួក គេ បាន ប្រើ ឧបករណ៍ នេះ ពី មុន ឬ អត់?
How do they diagnose what febrile illness a patient has? (prompt: whether mainly clinical diagnosis, what lab tests commonly used and available to aid dx of febrile patients)
6. តើអ្នកមានតេស្តអ្វីសម្រាប់នៅក្នុងមណ្ឌលសុខភាព? ឧ...អតិសុខុមទស្សជាដើម តើប្រើសម្រាប់ជម្ងឺអ្វីដែរ?
What tests do you have in the healthcare center (what kind of RDTs, any other tests e.g. microscopy? For what disease)
7. តើ នរណា ធ្វើ តេស្ត មន្ទីរ ពិសោធន៍ ទាំង នេះ (ប្រសិន បើ មាន) ? តើ មាន នរណា ម្នាក់ អាច ធ្វើ វា បាន ទេ ប្រសិន បើ បុគ្គល នោះ អវត្តមាន ?
Who performs these laboratory tests (if available)? Can someone else do it if the person is absent?</td></tr>
</table>

ការ បន្ថែម ការ វិនិច្ឆ័យ ថ្មី (ប្រើសំណួរគំរូសម្រាប់ឧបករណ៍នីមួយៗនេះ)<br>Addition of novel diagnostics<br>*(Use the sample questions for each of these tools)*	8. តើលោកអ្នកធ្លាប់ស្គាល់នូវ ឧបករណ៍នេះពីមុនដែរទេ?<br>(សូមបង្ហាញពីកញ្ចប់ដែលត្រូវបានណែនាំឲ្យប្រើ: multiplex RDT, dengue, and Malaria/CRP combo)?តើធ្លាប់ប្រហាក់ប្រហែលនិងឧបករណ៍នេះដែរទេ?(បង្ហាញឧបករណ៍RDT Dengue malaria/CRP combo}<br>Are you familiar with this kit before our workshop today (show them the kit that they were introduced to: multiplex RDT, dengue, and Malaria/CRP combo)?<br><br>9. តើ អ្នក យល់ឃើញ យ៉ាង ណា ក្នុង ការ ប្រើប្រាស់ ឧបករណ៍ នេះ បន្ទាប់ ពី ណែនាំរួច ?<br>How confident do you feel using this kit after the workshop?<br><br>10. តើ អ្នក មាន អារម្មណ៍ យ៉ាង ណា ដែរចំពោះ ការស្នើសុំ ឲ្យ ប្រើ ឧបករណ៍ នេះ (ពេញចិត្ត មិនពេញចិត្ត ងាយស្រួលប្រើ ពិបាក អាចគ្រប់គ្រងបាន) ? (Prompt: like or dislike; ease of use: easy/difficult/manageable/etc)How did you feel being asked to use the device? (Prompt: like or dislike; ease of use: easy/difficult/manageable/etc)<br><br>11. តើ អ្នក មាន អារម្មណ៍ ថា អ្នក បាន ទទួល ការ ណែនាំឬបណ្តុះ បណ្តាល គ្រប់ គ្រាន់ អំពី របៀប ប្រើ ឧបករណ៍ ទាំង នេះទេ?៖ multiplex RDT, dengue, និង Malaria/CRP combo? (បំផុសបន្ត៖ តើការបង្ហាត់បង្រៀនមានអត្ថប្រយោជន៍អ្វីខ្លះ? តើអ្នកចង់ឲ្យមានការបង្ហាត់បង្រៀនអ្វីទៀតនៅពេលអនាគតទេ?)<br>Do you feel that you have received adequate training on how to use these kits: multiplex RDT, dengue, and Malaria/CRP combo? (prompt: what aspects of training was useful? What more would you like to be trained in future?)<br><br>12. ជា រួម តើ អ្នក គិត ថា ឧបករណ៍ ទាំង នេះ អាច ត្រូវ បាន បញ្ចូល ក្នុង ការងារ របស់ អ្នក ឬ ទេ ? (បើអ្នកព្រមទទួលតើវាជាបន្ទុកនៅក្នុងការងាររបស់អ្នកដែរឬទេ? តើវាងាយស្រួលប្រើយ៉ាងដូចម្តេច? Probe: ការលំបាកបច្ចេកទេស, សុវត្ថិភាព, ពេលវេលាសម្រាប់ការធ្វើតេស្ត) ហេតុអ្វី?<br>Overall, do you think these kits can be (well) integrated in your work? (have you accepted them or are they still burden in your work? How easy is it to use? Probe: technical difficulties, safety, time taken for test) Why?

ការ កែ លម្អ សេវា<br>Product improvement	13. តើ អ្នក នឹង ដាក់ពិន្ទុប៉ុន្មានទៅលើការ ប្រើប្រាស់ ឧបករណ៍ថ្មីនេះ ?<br>How would you rate your overall experience using the device?<br><br>14. តើ អ្នក អាច ពន្យល់ ពី មូលហេតុ ដែល អ្នក បានផ្ដល់ពិន្ទុទៅលើ ឧបករណ៍ ថ្មីនេះ _ __? តើ អ្នក ចូលចិត្តលើ អ្វី ខ្លះ អំពី ឧបករណ៍ថ្មី នេះ ? តើ អ្នក មិន ចូល ចិត្ត អ្វី ខ្លះ អំពី ឧបករណ៍ ទាំង នេះ ?<br>Can you explain why you rated your experience as ___? What do you like and what do you not like?<br><br>15. តើ អ្នក គិត ថា គួរ តែ ធ្វើ ឲ្យ ប្រសើរ ឡើង យ៉ាង ដូចម្ដេចខ្លះ ជាមួយ ឧបករណ៍ នេះ ?<br>What do you think should be improved with the kit?<br>• ជាមួយនឹងវិធីដែលអ្នកទទួលដូចជាទឹកលាយ ឆ្នូត និងម៉ាស៊ីន<br>With the way you receive buffer, strips, and machine<br>○ i.e. Sufficient pipettes បំពង់គ្រប់គ្រាន់<br>• តើ អ្នក គិត យ៉ាង ណា អំពី កញ្ចប់ នោះ ?<br>What do you think about the packaging?<br>○ Of a new box of stripsនៃ ប្រអប់ ថ្មី នៃ ឆ្នូត<br>○ Packaging of pipettesការវេចខ្ចប់បំពង់បូម<br>○ Sealing of buffer

Use of novel diagnostics in future	16. តើ អ្នក គិត ថា ឧបករណ៍ ទាំង នេះ អាច ត្រូវ បាន យកមកប្រើ ដោយ បុគ្គលិក សុខ ភាព ផ្សេង ទៀត ដែល គ្មាន ការ ហ្វឹក ហាត់ តាម ស្តង់ដារ មួយ ដែរ ឬ ទេ ?<br>*(តើពួកគាត់អាចយកឧបករណ៍ជំងឺគ្រុនចាញ់ /CRP នេះនឹងអាចយកទៅប្រេលដោយបុគ្គលិកមណ្ឌលឬភ្នាក់ងារសុខភាពភូមិ ដោយគ្មានការណែនាំឬបណ្តុះបណ្តាលតាមស្តង់ដាប្រេលប្រាស់បានដែរឬទេ?)*<br>Do you think these kits can be used by health center workers or community health workers (including VMW), with or without a one day standardized hands-on training?<br>17. តើ អ្នក នឹង ផ្តល់ យោបល់ អ្វី ខ្លះ ដល់ បុគ្គលិក សុខភាពឬភ្នាក់ងារសុខភាពភូមិ ដែល មិន បាន ចូលវគ្គបណ្តុះបណ្តាល ដើម្បីឲ្យមានភាពងាយស្រួល អនុវត្ត ?<br>What would you suggest to other health center workers or community health workers (including VMW) on the kit's use, ease, practicalities?<br>18. តើ អ្នក នឹង ផ្តល់ យោបល់ អ្វី ខ្លះ ដល់ អ្នក បង្កើត គោលនយោបាយ/CNM អំពី ការ ប្រើប្រាស់ ឧបករណ៍នេះ? តើ អ្នក គិត ថា ឧបករណ៍ ទាំង នេះ អាច ត្រូវ បាន បន្ថែម ទៅ ក្នុងកញ្ចប់សេវា ប្រចាំ ថ្ងៃ របស់ បុគ្គលិក សុខ ភាព នៅ មណ្ឌល សុខ ភាព ដែរ ឬ ទេ ?<br>(ឬអ្វីទៅជាទស្សនៈរបស់អ្នក?)<br>What would you suggest policymakers/CNM about its use? Do you think these kits can be added into daily routine of health workers at health centre? (or what is your alternative opinion?)
Free opinion	19. តើអ្នកចង់ចែករំលែកជាមួយយើងនូវគំនិតណាមួយដែលខ្ញុំប្រហែលជាខកខានក្នុងការពិភាក្សាជាមួយអ្នកឬទេ?<br>Would you like to share with us any free opinion that I may have missed discussing with you?
The End	
