# Supplementary material for: The feasibility of novel point-of-care diagnostics for febrile illnesses at health centres in Southeast Asia: a mixed-methods study
Source: Trans R Soc Trop Med Hyg. 2023 Jun 15;117(11):788–96. doi: 10.1093/trstmh/trad036 (PMC10629948; doi:10.1093/trstmh/trad036)
Supplement: trad036_Supplemental_Files [file trad036_supplemental_files.zip › Supplementary data 3.pdf]

Structured observation checklist results

Dengue Duo

Five participants demonstrated the Dengue Duo (Table 1). The facilitators did not ask the dengue comprehension questions (Q0), hence this item could not be assessed. For Q2, four out of five participants did not collect enough blood and/or collected air bubbles in the pipette during the specimen collection for the NS1 test. Another participant collected too little blood for the NS1 test to be done. For Q5, one participant incorrectly transferred the blood for the serological arm of the test into the wrong well. For Q9, three out of four participants who were assessed did not have a complete understanding of how to interpret the antibody test result.

Table 1. Demonstration checklist results of the STANDARD™ Q Dengue Duo

STANDARD™ Q Dengue Duo steps	Participant				
	KK01	PT03	PT02	OC09	OC03
COMPREHENSION					
Q0: Dengue knowledge	N/A	N/A	N/A	N/A	N/A
PROCEDURE					
Q1: Asepsis and finger prick	2	2	2	2	2
Q2: Collect blood (NS1) with pipette	1	1	1	1	0
Q3: Transfer blood to NS1 device	2	2	2	2	N/A
Q4: Collect blood (AB test) with capillary tube	2	2	2	2	2
Q5: Transfer blood to AB device	2	2	1	2	2
Q6: Add buffer	2	2	2	2	2
READING AND INTERPRETATION					
Q7: Read results 15-20'	2	2	2	2	2
Q8: NS1 test interpretation	1	N/A	2	2	2
Q9: AB test interpretation	1	N/A	1	2	1

Table 1 gives the assessment results of participants performing the STANDARD™ Q Dengue Duo test. Each step of the procedure was graded with 0 (not done), 1 (done imperfectly or requiring help), or 2 (done perfectly).

Malaria/CRP Duo

Three participants demonstrated the Malaria/CRP Duo (Table 2). All had good basic comprehension of malaria and CRP (Q0). All performed the test correctly, except one who conducted Q5 imperfectly by pressing the bulb of the capillary tube and collecting too much blood. All participants could interpret the malaria and CRP results correctly (Q10, Q11).

Table 2. Demonstration checklist results of the STANDARD™ Q Malaria/CRP Duo

STANDARD™ Q Malaria/CRP Duo steps	Participant score		
	BR04	BR07	CR01
COMPREHENSION			
Q0: Malaria and CRP knowledge	2	2	2
PROCEDURE			
Q1: Asepsis and finger prick	2	2	2
Q2: Collect blood with the inverted cup (malaria)	2	2	2
Q3: Transfer blood to the specimen well of malaria device	2	2	2
Q4: Add buffer (malaria)	2	2	2
Q5: Collect blood (CRP) with capillary tube	2	2	1
Q6: Put blood with assay diluent	2	2	2
Q7: Mix specimen by pipetting	2	2	2
Q8: Transfer specimen to CRP device	2	2	2
READING AND INTERPRETATION			
Q9: Read results at 15-20'	2	2	2
Q10: Malaria test interpretation	2	2	2
Q11: CRP test interpretation	2	2	2

Table 2 gives the assessment results of participants performing the STANDARD™ Q Malaria/CRP Duo test. Each step of the procedure was graded with 0 (not done or no comprehension), 1 (done imperfectly or requiring help, or imperfect comprehension), or 2 (done perfectly or perfect comprehension).

DPP® Fever Panel II Asia Antigen

Four participants performed the Fever Panel II Asia Antigen test (Table 3). One participant needed help from their colleague to do a finger prick on the patient (Q1). One participant had to be guided on which well to add the sample buffer (Q4). Two participants needed help in setting up the reader (Q8). Two participants needed help putting in the test device into the reader (Q9).

Table 3. Demonstration checklist results of the DPP® Fever Panel Asia II Antigen

DPP® Fever Panel II Asia Antigen steps	Participant			
	KC01	KC05	SK02	SK06
Q1: Asepsis and finger prick	2	2	2	1
Q2: Collect blood with capillary tube	2	2	2	2
Q3: Transfer blood to DPP Ag Well1	2	2	2	2
Q4: Add sample buffer (Ag) to Well1	2	2	1	2
Q5: Start timer	2	2	2	2
Q6: At 5', add buffer to DPP Ag Well2	2	2	2	2
Q7: Read results at 20-25'	2	2	2	2
Q8: Set up reader with RFID Ag card	1	2	2	1
Q9: Put in test device into reader	2	2	1	1

Table 3 gives the assessment results of participants performing the DPP Fever Panel II Asia Antigen test. Each step of the procedure was graded with 0 (not done), 1 (done imperfectly or requiring help), or 2 (done perfectly).

DPP® Fever Panel II Asia Antibody

Four participants practiced the Fever Panel II Asia Antibody test (Table 4). One participant did not prepare the sample buffer in the sample vial before pricking the finger of the patient (Q1). One participant transferred the blood into the sample vial imperfectly, releasing the blood to the wall of the vial (Q4). Two participants produced air bubbles when mixing the specimen by pipetting (Q5). Two participants needed guidance on operating the reader (Q11).

Table 4. Demonstration checklist results of the DPP® Fever Panel Asia II Antibody

DPP® Fever Panel II Asia Antibody steps	Participant			
	KL06	KL05	TS05	TS08
Q1: Prepare the sample buffer by putting 5 drops into the sample vial	2	1	2	2
Q2: Asepsis and finger prick	2	2	2	2
Q3: Collect blood with capillary tube	2	2	2	2
Q4: Transfer blood to sample vial	2	1	2	2
Q5: Mix specimen by pipetting	2	1	2	1
Q6: Collect specimen with transfer pipette	2	2	2	2
Q7: Transfer specimen to DPP Ag Well1	2	2	2	2
Q8: Start timer	2	2	2	2
Q9: At 5', add running buffer to DPP Ag Well2	2	2	2	2
Q10: Read results at 20-25'	2	2	2	2
Q11: Set up reader with RFID IgM card	1	1	2	2
Q12: Put in test device into reader	2	2	2	2

Table 4 gives the assessment results of participants performing the DPP Fever Panel II Asia Antibody test. Each step of the procedure was graded with 0 (not done), 1 (done imperfectly or requiring help), or 2 (done perfectly).
