# Supplementary material for: The feasibility of novel point-of-care diagnostics for febrile illnesses at health centres in Southeast Asia: a mixed-methods study
Source: Trans R Soc Trop Med Hyg. 2023 Jun 15;117(11):788–96. doi: 10.1093/trstmh/trad036 (PMC10629948; doi:10.1093/trstmh/trad036)
Supplement: trad036_Supplemental_Files [file trad036_supplemental_files.zip › Supplementary data 4.pdf]

Table S1. Participant list

Health Center	No	Sex	Age	Education
Dengue Duo				
**Kosh Kralor**, Battambang Province, 8 participants	KK01	F	31	Secondary midwife
	KK02	F	30	Secondary midwife
	KK03	F	31	Secondary midwife
	KK04	F	32	Secondary midwife
	KK05	F	33	Secondary midwife
	KK06	F	36	Secondary midwife
	KK07	M	42	Primary nurse
	KK08	F	30	Secondary midwife
**Prey Tralach.** Battambang Province, 7 participants	PT01	M	57	Primary nurse
	PT02	F	38	Primary midwife
	PT03	M	26	Primary nurse
	PT04	M	50	Secondary nurse
	PT05	M	35	Lab.technician
	PT06	M	33	Primary nurse
	PT07	M	52	Primary nurse
**O'Chra,** Pailin Province, 9 participants	OC01	M	38	Secondary nurse
	OC02	F	46	Primary midwife
	OC03	F	32	Primary midwife
	OC04	F	31	Primary midwife
	OC05	F	30	Primary midwife
	OC06	M	49	Primary nurse
	OC07	M	33	Primary nurse
	OC08	F	31	Primary midwife
	OC09	M	46	Lab.technician
Malaria/CRP Duo				
**Boeung Run,** Battambang Province, 7 participants	BR01	M	59	Secondary Nurse
	BR02	M	46	Secondary Nurse
	BR03	M	48	Primary nurse
	BR04	M	32	Secondary Nurse
	BR05	M	36	Secondary Nurse
	BR06	F	28	Secondary Nurse
	BR07	M	30	Secondary Nurse
**Chorrk Roka,** Battambang Province, 8 participants	CR01	F	32	Primary midwife
	CR02	F	50	Secondary midwife
	CR03	F	53	Primary midwife
	CR04	F	41	Primary midwife
	CR05	F	42	Primary midwife
	CR06	F	42	Primary midwife
	CR07	M	40	Primary midwife
	CR08	F	41	Primary midwife
DPP Antigen				
**Krachab,** Pailin Province, 5 participants	KC01	M	37	Secondary Nurse
	KC02	F	29	Secondary midwife
	KC03	F	27	Secondary midwife

Health Center	No	Sex	Age	Education
	KC04	F	25	Secondary midwife
	KC05	F	34	Secondary midwife
**Soun Koma,** Pailin Province, 7 participants	SK01	M	53	Primary nurse
	SK02	M	45	Primary nurse
	SK03	F	30	Secondary midwife
	SK04	F	43	Secondary Nurse
	SK05	M	31	Primary nurse
	SK06	M	35	Secondary nurse
	SK07	F	33	Primary nurse
DPP Antibody				
**Kampong Lpov,** Battambang Province, 8 participants	KL01	F	60	Secondary Nurse
	KL02	M	50	Primary nurse
	KL03	F	30	Secondary nurse
	KL04	M	45	Secondary nurse
	KL05	M	55	Secondary nurse
	KL06	F	41	Primary nurse
	KL07	F	42	Primary nurse
	KL08	M	54	Primary nurse
**Tasanh,** Battambang Province, 10 participants	TS01	F	27	Secondary midwife
	TS02	F	29	Secondary midwife
	TS03	M	55	Secondary nurse
	TS04	F	40	Primary nurse
	TS05	M	45	Secondary nurse
	TS06	M	34	Secondary nurse
	TS07	M	43	Secondary nurse
	TS08	F	42	Primary nurse
	TS09	F	40	Primary nurse
	TS10	F	59	Secondary midwife

ANC: antenatal care, IMCI: integrated management of childhood illnesses, TB: tuberculosis, OPD: outpatient department; Primary midwife: midwife with one year midwifery education; Secondary midwife: midwife with three years midwifery education; Primary nurse: nurse with one year training; Secondary nurse: nurse with three years of training (Registered Nurse).
