# Supplementary material for: The feasibility of novel point-of-care diagnostics for febrile illnesses at health centres in Southeast Asia: a mixed-methods study
Source: Trans R Soc Trop Med Hyg. 2023 Jun 15;117(11):788–96. doi: 10.1093/trstmh/trad036 (PMC10629948; doi:10.1093/trstmh/trad036)
Supplement: trad036_Supplemental_Files [file trad036_supplemental_files.zip › Supplementary data 5.pdf]

Table S2. Quotes on technical difficulties

Subtheme	Dengue Duo	Malaria/CRP	DPP®
Blood sampling			
	Because we need more quantity of blood, we cannot take it in a hurry. If we take the blood sample carefully like we do, I can use one blood sample for the three-item test. So, we cannot pump more blood within a shorter period of time. (Kosh Kralor)	and as for the tube, it can be problematic for them where the pipetting of the blood volume can be too much or it can be difficult to get a sufficient amount volume. Even though we have another way of obtaining blood with pressing and depressing or by massaging, sometimes it still cannot reach the required amount. (Boeung Run)	
	As you could see the test just now, it was hard to pump blood for test. So, getting blood for test is not good, and the squeezing of blood is not good either. It causes us to take more time and consumes more needles.<br>(Prey Tralach)	so we must take three tubes of blood and in my opinion it is also somewhat difficult. But this difficulty, with us practising it on a daily basis, will turn out to be better. But for the newcomers, it becomes somewhat harder because firstly it is hard with pipetting the blood; secondly an inadequate amount of collection; and thirdly with the children who are restless and we collect an inadequate amount of blood and that is also a challenge. (Boeung Run)	
	Well, 100 micro-litre tubes make a little hard to get enough blood for the test. It relates to technical issue, and it is hard for us to make a request [to the patient, to do more than one finger prick to get blood]. Technically it is not correct until the tubes are 10 micro-litres [filled to the specified amount, whether 10 or 100 microlitres]. Using 100 micro-litre tubes is hard for us to get enough blood because the patients might get bored with the physicians. The physicians are not bored with the patients. Some children are scared, and they do not allow us to take their blood. We need to take the blood twice to prevent a lack of blood, so they get scared and do not allow us to take their blood again. Children get panicked because we need to squeeze for the blood to come out; that is why they become frightened.<br>(Prey Tralach)		

Subtheme	Dengue Duo	Malaria/CRP	DPP®
	No matter how hard we squeeze, the amount of blood we get is limited and getting blood out for the test is a little difficult as well. (O'Chra)		
	One more thing I would like to contribute; if possible, we should take less blood than that for the antigen test, just enough so that the test works. But if we take less [than100 microlitres], the blood may not be enough to do the test, so what do the scientists think if we take less blood than that? (O'Chra)		
	but for the big one [the NS1], if we are careless, it might have air inside the pipette which results in less amount of blood. And if we squeeze the pipette bulb slowly to avoid air bubbles, it is alright; and if the air is about to go inside, we release the air bubble quickly. (O'Chra)		
	For the application of this test, it is not a problem, just a bit hard at the beginning for getting more blood If it needed less blood, it would be easy. (O'Chra)		
Complexity/mix-ups			
	If it could be done only one time together with only one result, it would be more convenient. (Kosh Kralor)	our staff members think that the testing will be easy. But for the professionals, they think that it may cause confusion because it appears to have multiple steps as well. (Boeung Run)	about buffer liquid, please use different colours because sometimes we only read the letters, and sometimes we can recognise the colour of the bottle lids. … We can print Spec 1 (specimen 1), Spec 2 (specimen 2) because there is a buffer sample inside. We can print abbreviated letters on the buffer sample. It is easier. … The test is already numbered 1 or 2, but the liquid is not numbered, except in English script. If possible, both the test and liquid should be numbered. That side is already printed with English script, so English script should also be written on this side. (Krachab)
	It would be better if we could have a bit deeper well to store blood for the antigen test because the colour [of the kit] is clear, shallow, and small. (Kosh Kralor)	I think that if the test is done by dropping a blood sample and buffer liquid in their respective spots then it is easy as there are two wells. But this test has only one well while the other test has two wells and sometimes they may get confused and	I think the packaging is already good, but I would like a modification on the mixing liquid, like they already said. (Krachab)

Subtheme	Dengue Duo	Malaria/CRP	DPP®
		place the blood on the well for the solution and vice versa. (Boeung Run)	
		The second thing is that for the placement of buffer and the blood. We have three blood sampling tool, one for malaria, but the other two we don't really know. But if we have participated in this training session, we would know that one tube is to collect the blood, and the other tube is for mixing the blood with the buffer and transferring the mix into the well. So, there are a lot of challenges for this issue too. But if we have one test for detection of only one infection then it is not confusing, [but in this kit] there are multiple materials with differing functions. (Boeung Run)	regarding the sample buffer and running bufer, I would like the company to make it different; otherwise, it would be easy for our team to be confused between sample buffer and running bufer. So, please ensure they are not confused. For example, it can be labelled whatever number on this lid, so it would be easier than normal. (Soun Koma)
		Yes, looking at the multiple wells, sometime we might put the blood in the wrong well. (Chorrk Roka)	I think if there was translation [of the labels] into Khmer, it would be great.<br>(Kmpong Lpov)
			I fear that I am confused with this test. Me too, like [another participant] said earlier whether it is possible to make the lid for one item in a different colour like blue or red. Sometimes we can read the text but when we are so busy it is possible that we catch the wrong things. (Tasanh)
			This [reader] machine is easy but this card is confusing because there are two kinds of that card (Antibody card and Antigen card). (Tasanh)
			For the card, I think that we can write it in Khmer language on one side and English on the other side for those who do not know the language to understand as well. Some of our staff members may have some limitations with understanding us placing English text on one side and Khmer text on the other side, and with the number placed to specify what they are. (Tasanh)
			I fear confusion in the placement of the solution drop and one more thing, in terms of setting the minutes; I don't remember whether it is from 5 minutes to 15 minutes. But if possible, you can help write it down on the bottle or the paper or

Subtheme	Dengue Duo	Malaria/CRP	DPP®
			give some kind of instruction to be easy to understand. (Tasanh)
Waiting time			
			It would be good if we could reduce the duration because we wait up to 20 minutes. And one more thing, we need to do a lot of work in the health centre, not only one. Sometimes there are many patients, and some days there were many cases of illness. One machine takes a lot of time, up to 20 minutes, so it would take the whole morning if there were three patients. So, it would be good if the duration can be shortened. (Krachab)
			A: For me, the disadvantage is the waiting time for the result.<br><br>Q: Does it take too long?<br><br>A: Well, the duration is not too long, but there are many steps, and for me, I do not have any issue because I am a medical staff, so the problem is for the patients who need to wait for a longer time. (Soun Koma)
			The important thing is the package is solid and the wait time should be faster like from 15 minutes to 10 minutes or from 5 minutes to 3 minutes because it saves time. (Kmpong Lpov)
Interpretation of test			
			I do not understand the meaning of Normal or Abnormal. Like he requested, we have already practised the test, but we only know 2, 3, and 4 (numbers); however, we do not know whether or not there is a disease. Thus, I would like a clearer explanation and request more training courses. (Krachab)
			A: Another consequence is that, we do not know how to conclude [what disease the patients are infected with] because we do not know the result yet.

Subtheme	Dengue Duo	Malaria/CRP	DPP®
			Q: OK, that is correct. It means it does not have guidelines yet.<br><br>A: If required by the ministry with proper instructions, when we know the result, it will be easy for me to inform the patient what illness they really have—chikunguya, malaria, or dengue, or what parasite, bacteria or virus causes the illness.<br><br>Q: Yes, thank you very much. Your difficulty is that there are no clear guidelines on whether and how many viruses are considered positive, and how many viruses are considered negative.<br>(Soun Koma)
			A: So, it does not show red, does it? For example, how long does it take before it shows red?<br><br>…<br><br>Q: So, you mean it is positive if this number becomes red. You want this machine to show that, if the number becomes red, it is problematic, so we consider it positive, but for the numbers, let the Ministry of Health make a decision.<br><br>A: We cannot set it unless there are guidelines or instructions to follow. However, if [the significant results are shown in] red [colour], we understand that it is problematic. It does not show red until it gains [pathogens] at the right levels so as to inform us that there is malaria, and we will start treatment like malaria, for instance.<br>(Soun Koma)
			the reading of the result is not so understandable and the demonstration done earlier is per memory and it is not very clear. (Tasanh)
