# Supplementary material for: The feasibility of novel point-of-care diagnostics for febrile illnesses at health centres in Southeast Asia: a mixed-methods study
Source: Trans R Soc Trop Med Hyg. 2023 Jun 15;117(11):788–96. doi: 10.1093/trstmh/trad036 (PMC10629948; doi:10.1093/trstmh/trad036)

STANDARD™ Q
Malaria/CRP Duo, Dengue Duo

Malaria/CRP Duo

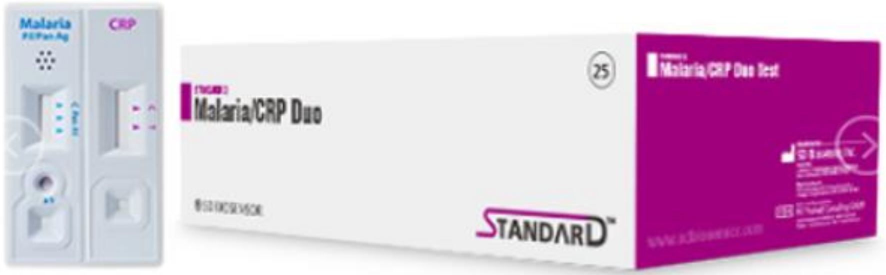

- Lateral flow test: detects CRP and Malaria (2 devices side-by-side)
- Malaria: *P. falciparum* & PAN (*P. vivax, P. ovale, P. malariae*)
- CRP: Detection limit 20mg/L

Malaria/CRP Duo – Doing the test

1. Check expiry data
2. Open foil pouch and check test device and silica pack (must be yellow).

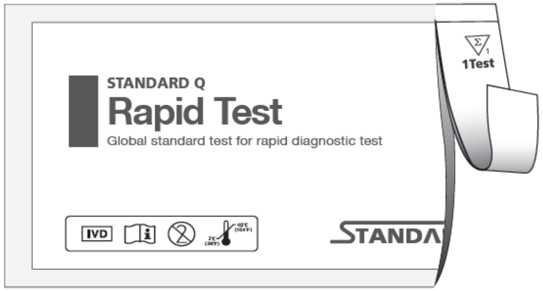

<Foil pouch>

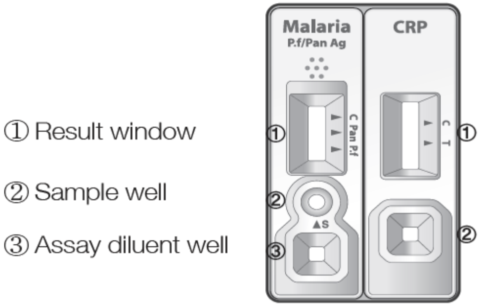

<Test device>

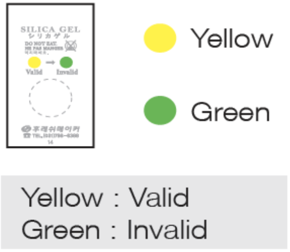

<Silica gel>

3. Wipe finger with an alcohol wipe and prick with a lancet.

Malaria/CRP Duo – Doing the test

Malaria part of the test

1. Collect the blood with the blood sample device (5ul)

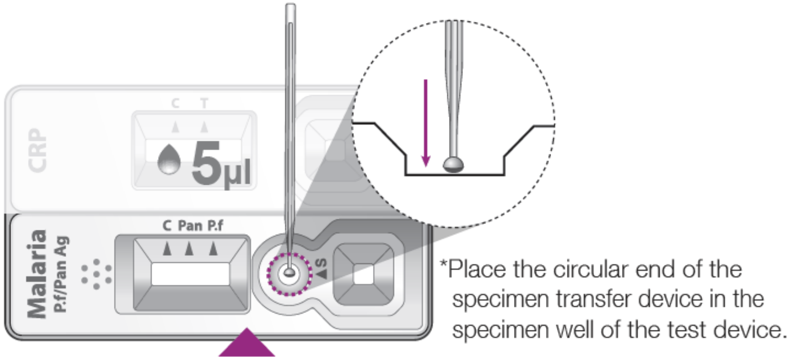

2. Add 3 drops of buffer to the square well of the test device

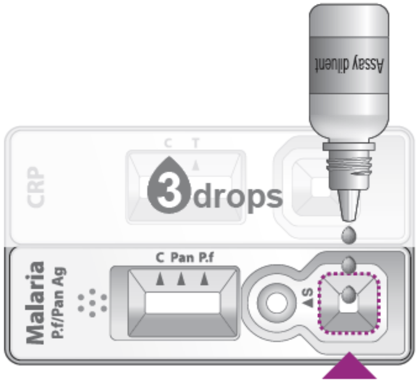

Malaria/CRP Duo – Doing the test

CRP part of the test

1. Collect the blood with the Ezi tube collector (10ul). Fill to black line

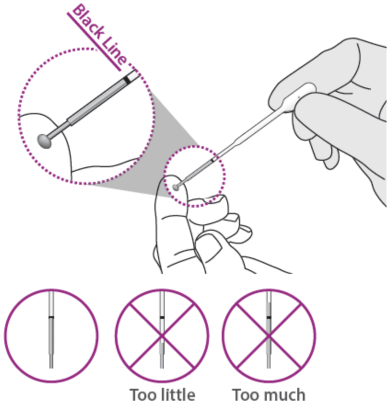

Malaria/CRP Duo – Doing the test

CRP part of the test

2. Add blood to the assay diluent, dispose of Ezi tube (10ul)

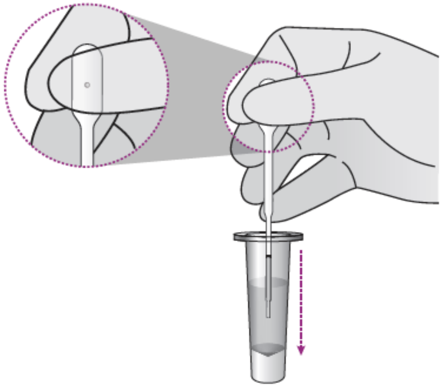

3. Use dropper (100ul) to mix the specimen. Carefully press and release 6-8 times.

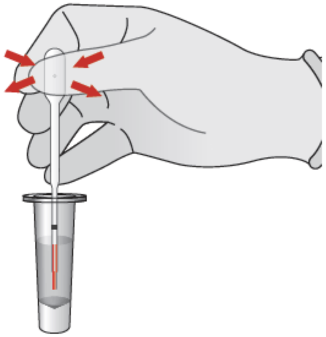

Malaria/CRP Duo – Doing the test

CRP part of the test

4. Collect ALL specimen and add it ALL to the sample well.

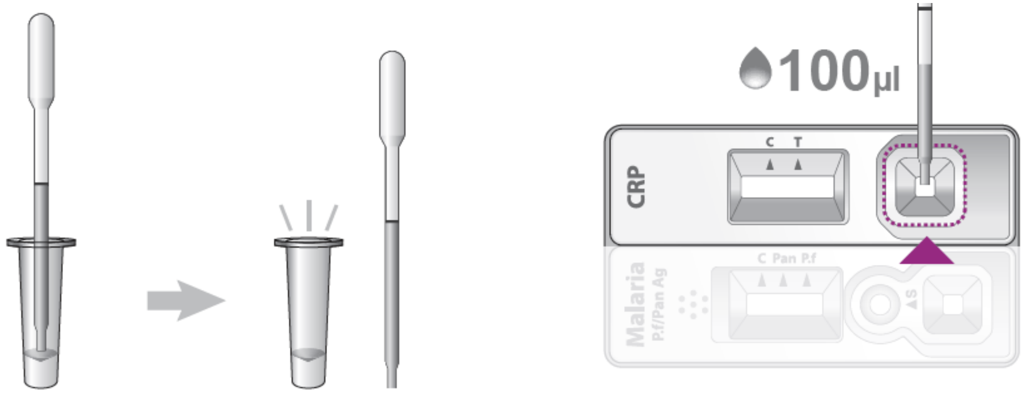

Malaria/CRP Duo – Reading the result

Read both the test results at 15-20 mins

Do not read after 20 mins

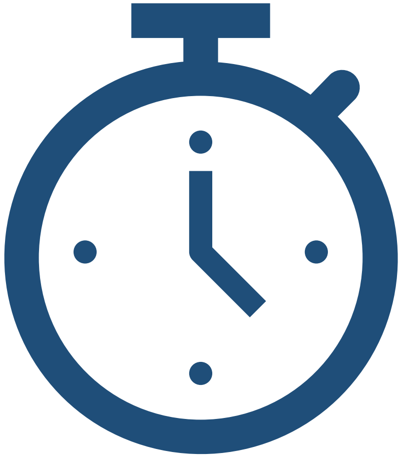

MORU Tropical Health Network
wellcome
UNIVERSITY OF OXFORD

Malaria/CRP Duo – Reading the result

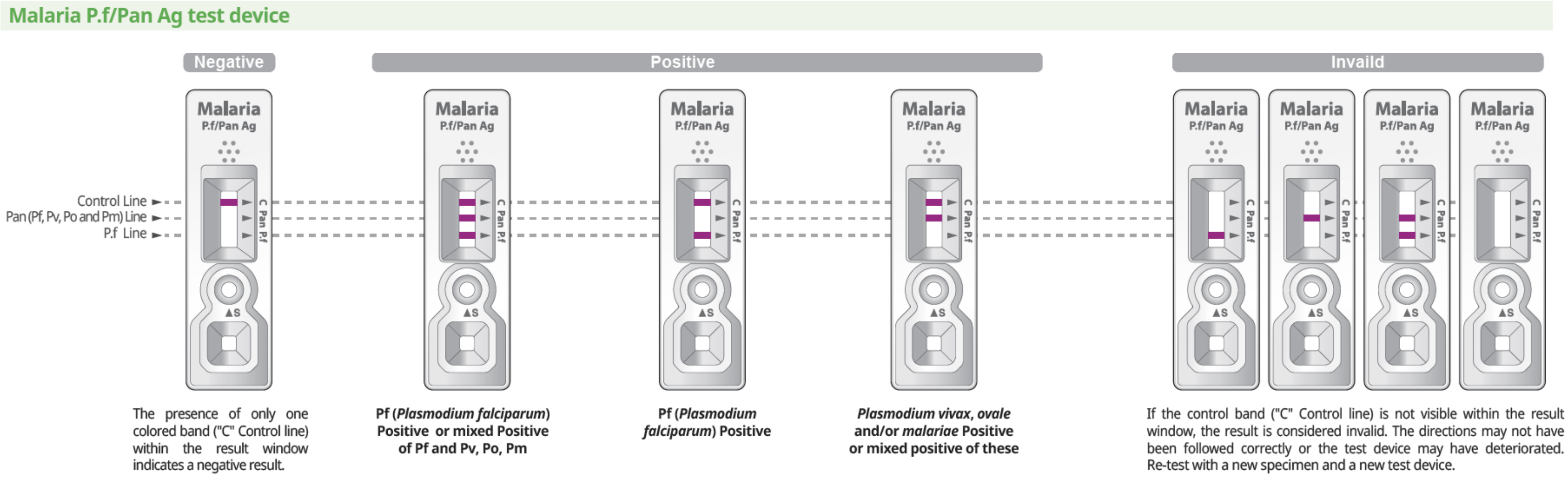

Malaria/CRP Duo – Reading the result

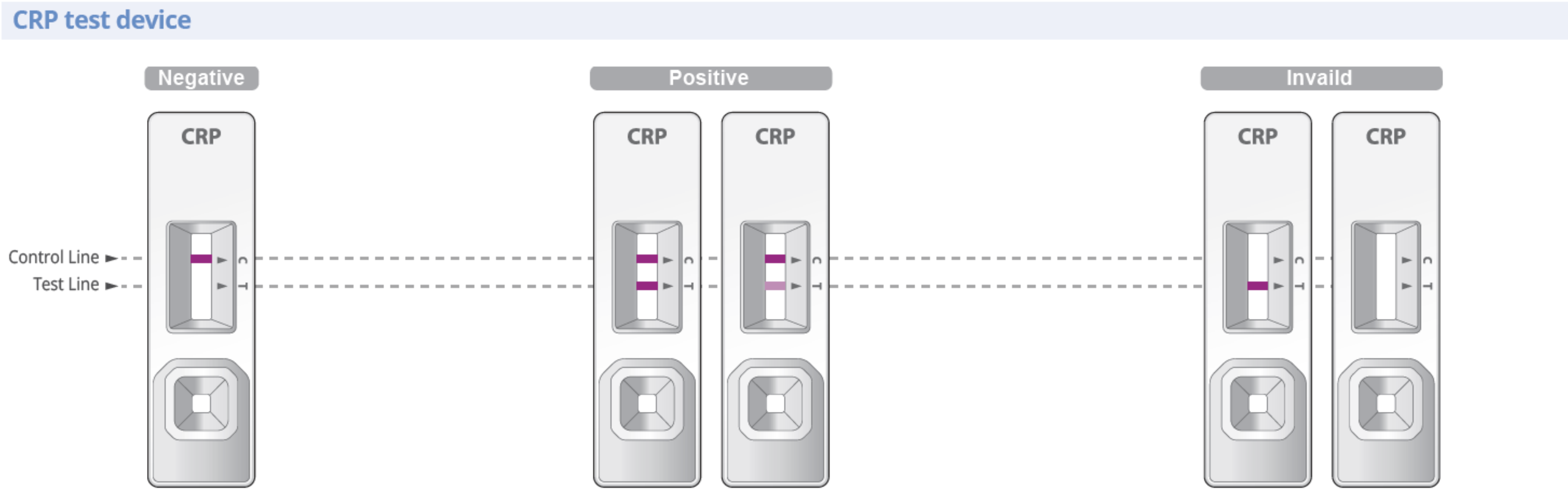

CRP level is lower than 20mg/L

CRP level is above 20mg/L

If the control band ("C" Control line) is not visible within the result window, the result is considered invalid. The directions may not have been followed correctly or the test device may have deteriorated. Re-test with a new specimen and a new test device.

Dengue Duo

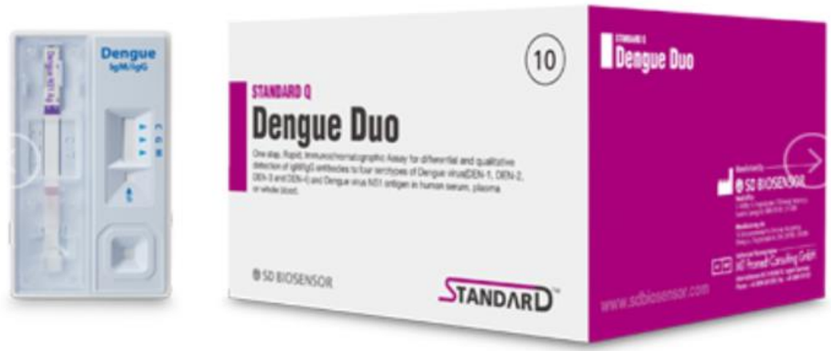

- One-step assay that detects dengue antigens and antibodies
- 2 test devices side-by-side

Dengue Duo – Doing the test

1. Check expiry data
2. Open foil pouch and check test device and silica pack (must be yellow).

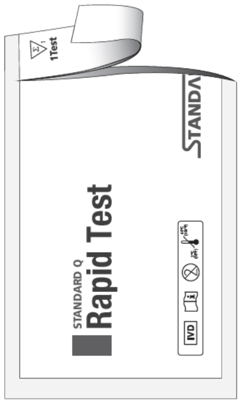

<Foil pouch> / <Emballage d'aluminium>

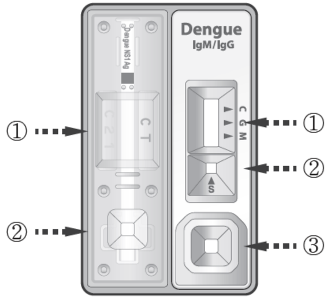

<Test device> / <Dispositif de test>

[EN] ① Result window
② Sample well
③ Assay diluent well

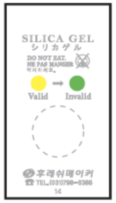

Yellow / Jaune / Amarillo / Válido

Green / Vert / Verde / Inválido

[EN] Yellow : Valid / Green : Invalid

3. Wipe finger with an alcohol wipe and prick with a lancet.

Dengue Duo – Doing the test

NS1 test (Antigen test)

1. Using sample collector, add blood to sample device (100ul)

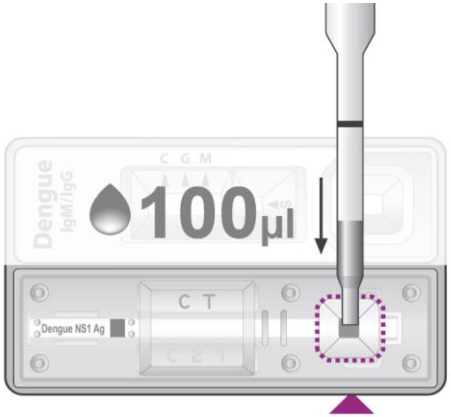

Dengue Duo – Doing the test

Dengue IgM/IgG test (antibody)

1. Using Ezi tube collector, add blood to the sample device (10ul)

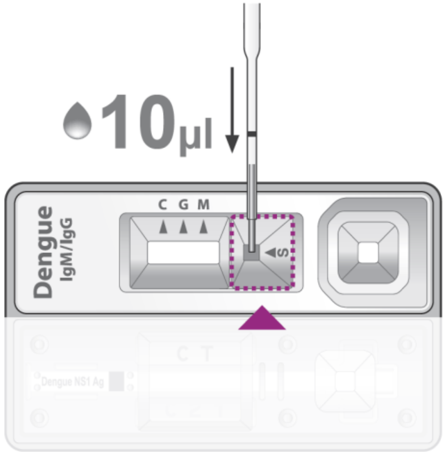

2. Add 3 drops of buffer to the square well of the test device

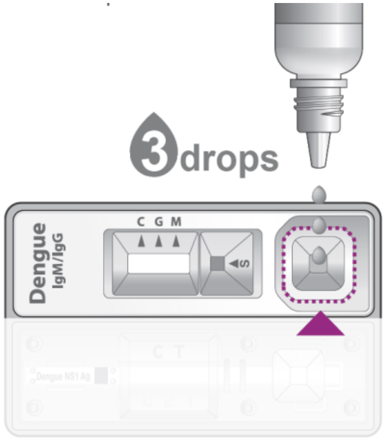

Dengue Duo RDT – Reading the result

Read both the test results at 15-20 mins

Do not read after 20 mins

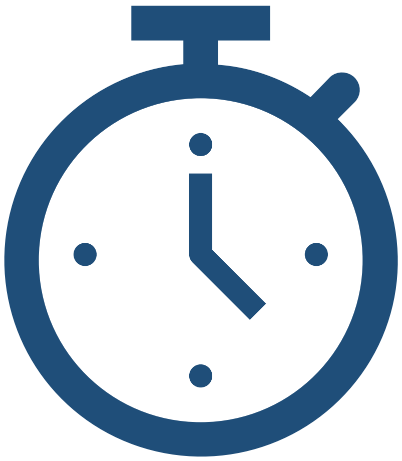

Dengue Duo RDT – Reading the result

Dengue NS1

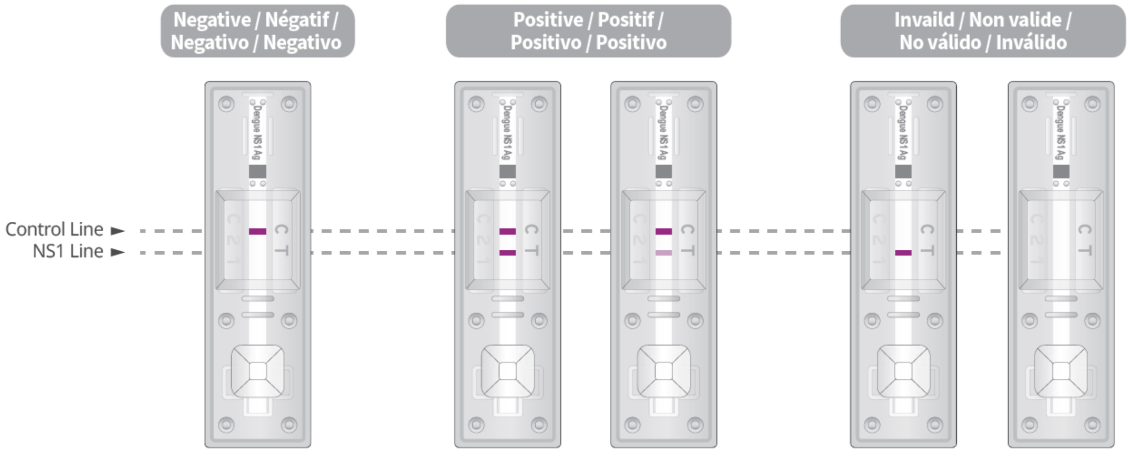

[EN] Dengue NS1 Negative
[FR] Négatif pour l'antigène NS1 de la dengue
[ES] Dengue NS1 negativo
[PT] Dengue NS1 Negativo

[EN] Dengue NS1 Positive
[FR] Positif pour l'antigène NS1 de la dengue
[ES] Dengue NS1 positivo
[PT] Dengue NS1 Positivo

[EN] Re-test with a new test device.
[FR] Retester avec un nouveau dispositif de test.
[ES] Repita la prueba con un nuevo dispositivo de prueba.
[PT] Teste novamente com um novo dispositivo de teste.

Dengue Duo RDT – Reading the result

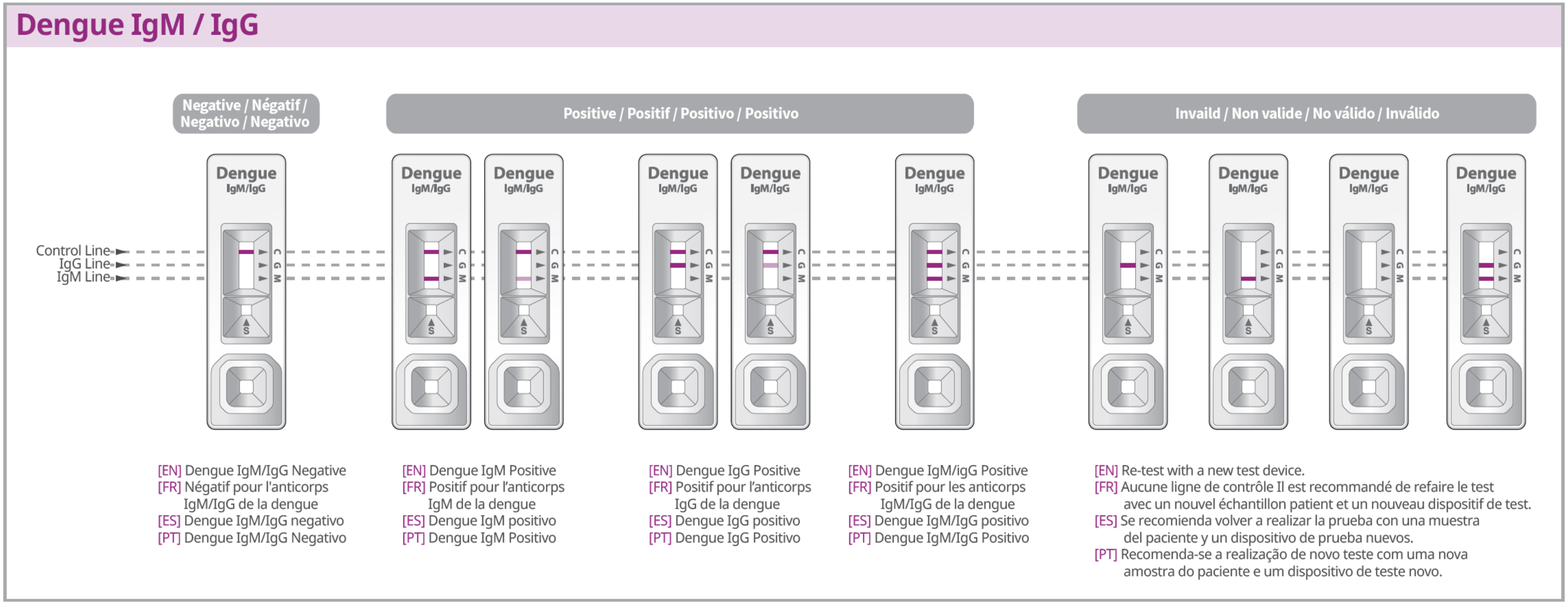

Supplement: trad036_Supplemental_Files [file trad036_supplemental_files.zip › Supplementary data 6.pdf]
