# Supplementary material for: The feasibility of novel point-of-care diagnostics for febrile illnesses at health centres in Southeast Asia: a mixed-methods study
Source: Trans R Soc Trop Med Hyg. 2023 Jun 15;117(11):788–96. doi: 10.1093/trstmh/trad036 (PMC10629948; doi:10.1093/trstmh/trad036)
Supplement: trad036_Supplemental_Files [file trad036_supplemental_files.zip › Supplementary data 7.pdf]

DPP® Fever Panel II Asia Antibody and Antigen

DPP® Fever Panel II Asia

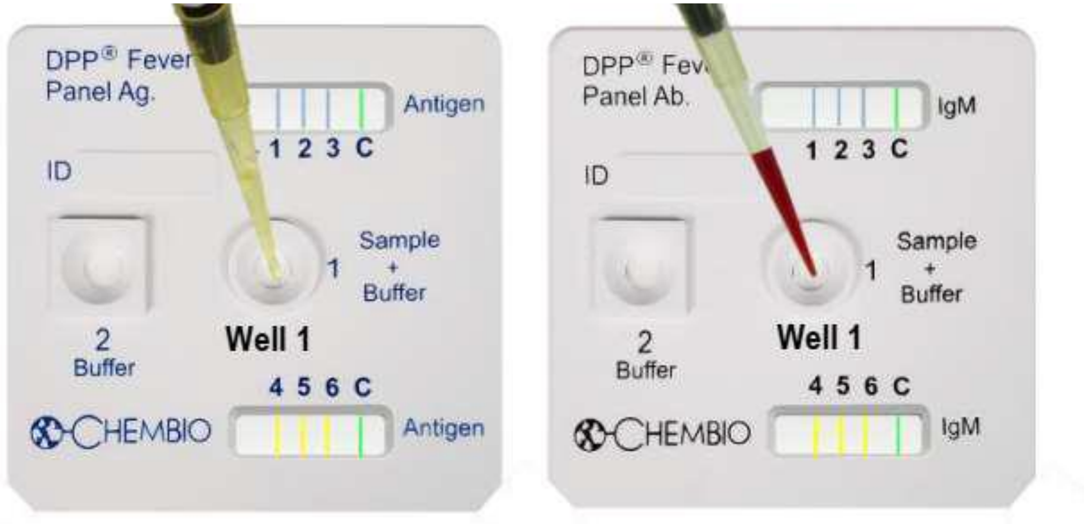

- Two kinds
- DPP Fever Panel Antigen System
  - Detects the virus/bacteria/parasite
- DPP Fever Panel IgM System
  - Detects the immune response to the virus/bacteria/parasite

MORU Tropical Health Network
wellcome
UNIVERSITY OF OXFORD

DPP® Fever Panel II Asia

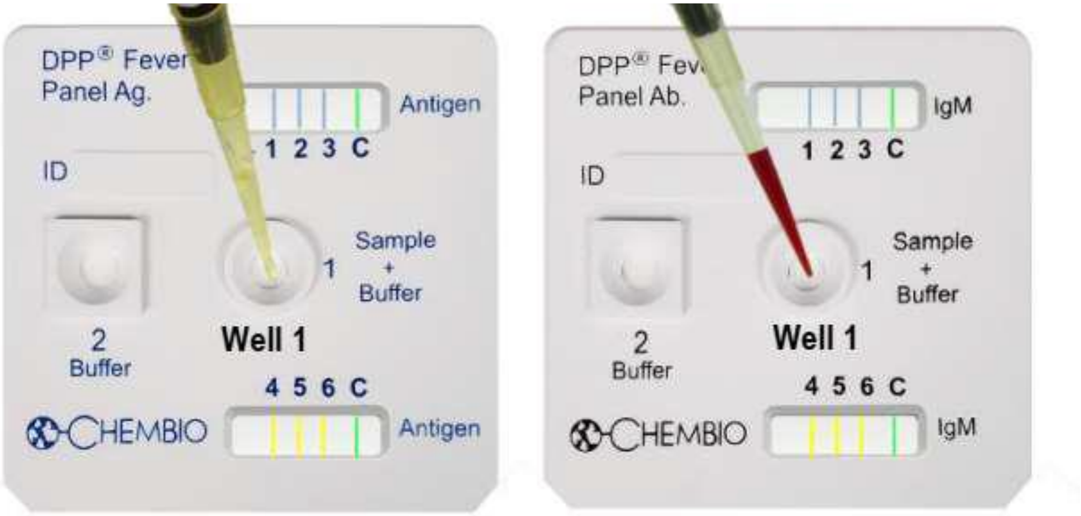

- DPP Fever Panel Antigen System
  - Chikungunya, Dengue, Zika, pan malaria, Pf
  - Burkholderia (melioidosis)
- DPP Fever Panel IgM System
  - Chikungunya, Dengue, Zika
  - Leptospira, Orientia tsutsugamushi (scrub typhus), Rickettsia typhi (murine typhus)

DPP® Antigen system

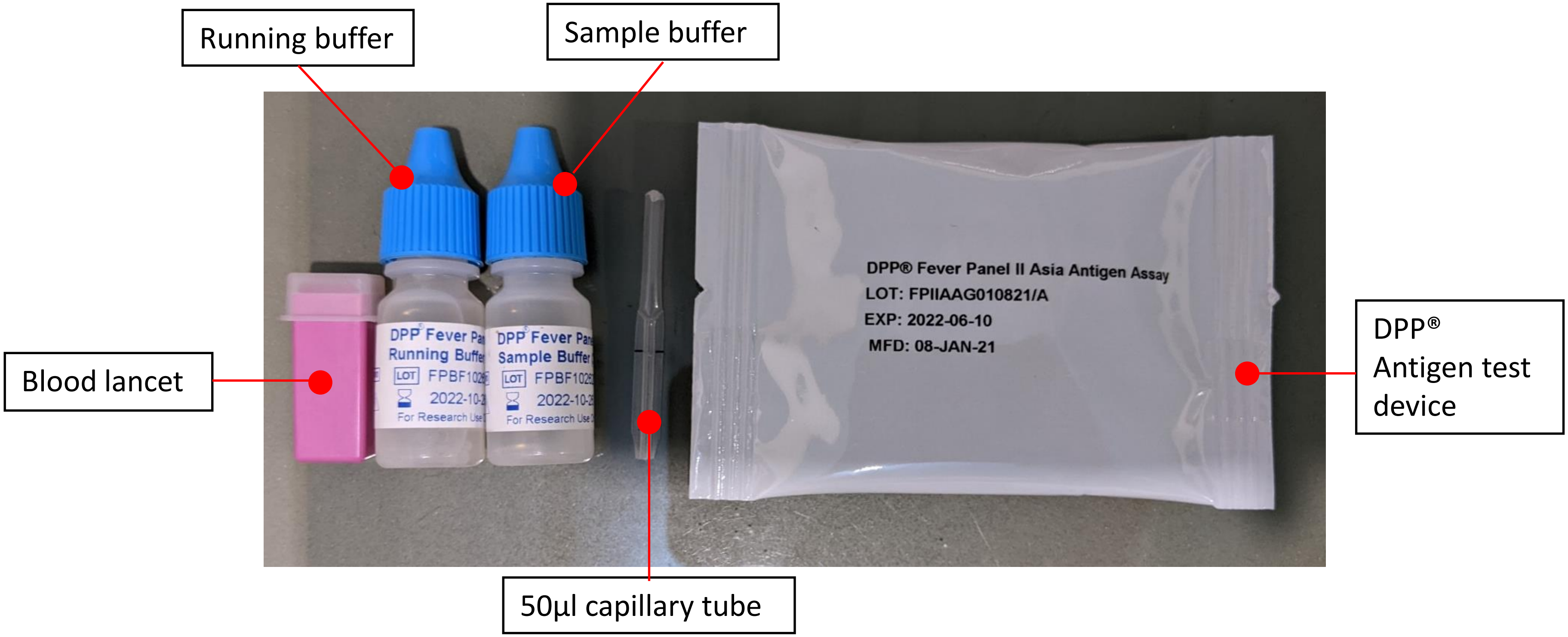

Not supplied in the box:
- Gloves
- Alcohol wipe
- Sharps disposal
- Timer

DPP® Antigen system

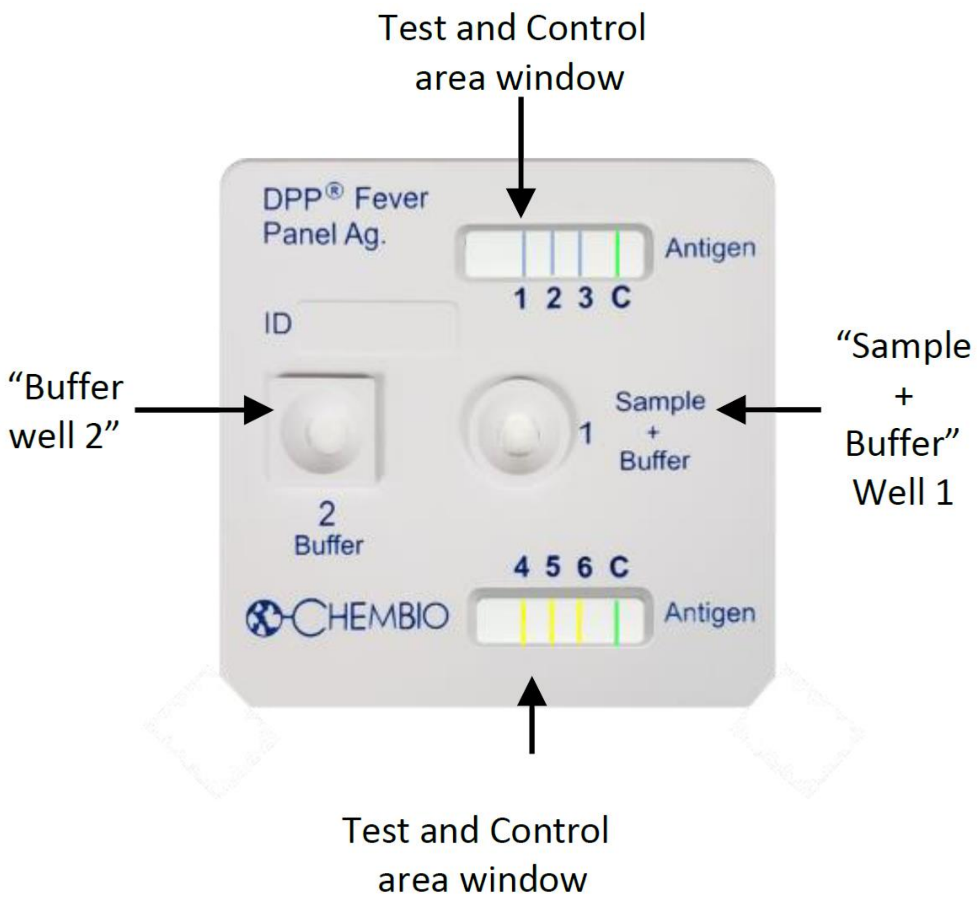

Antigen Detection

1. Chikungunya
2. Malaria pLDH
3. Dengue NS1
4. Zika
5. Malaria HRPII
6. Burkholderia p. CPS (melioidosis)

DPP® Antigen system: doing the test

1. Collect the blood by touching it with the open end of the 50µl capillary tube. Hold it horizontally and let blood seep up to the black line.
   Do not squeeze the tube bulb.

Figure 1: Capillary Pipette

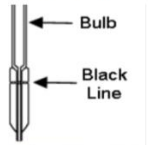

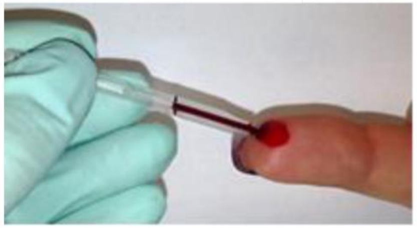

Figure 2: Capillary Pipette filled with Blood

2. Release all the blood onto the "Sample+Buffer Well 1" by squeezing the tube bulb.

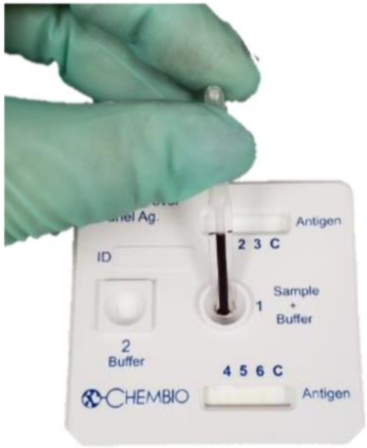

DPP® Antigen system: doing the test

3. Add 4 drops of the DPP® Antigen Sample Buffer into the “Sample+Buffer Well 1” (same well as the blood).

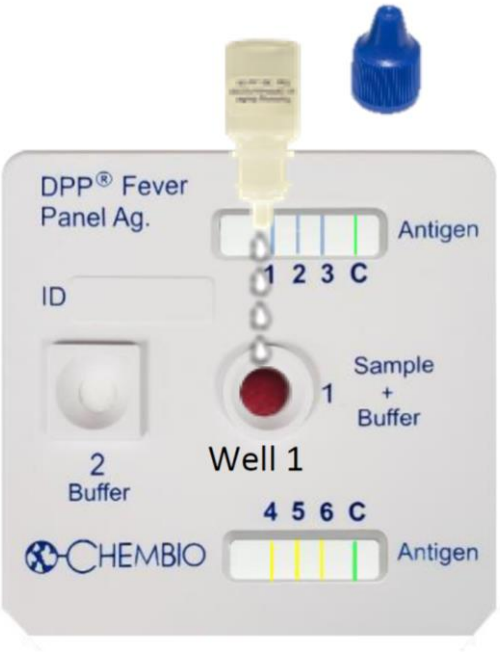

4 drops

4. Start a timer immediately.

MORU Tropical Health Network
wellcome
UNIVERSITY OF OXFORD

DPP® Antigen system: doing the test

5. At **5 minutes**, add 12 drops the DPP® Antigen Running Buffer to the “Buffer Well 2".

6. At **20-25 minutes**, read the results with the DPP® Micro Reader 2. Do not read after 25 minutes.

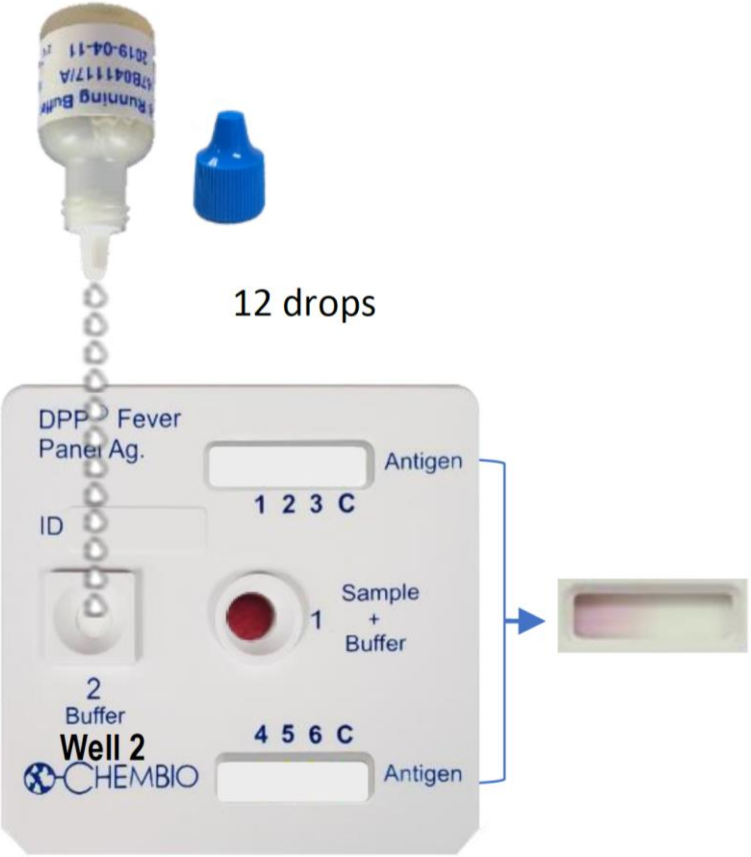

DPP® IgM system

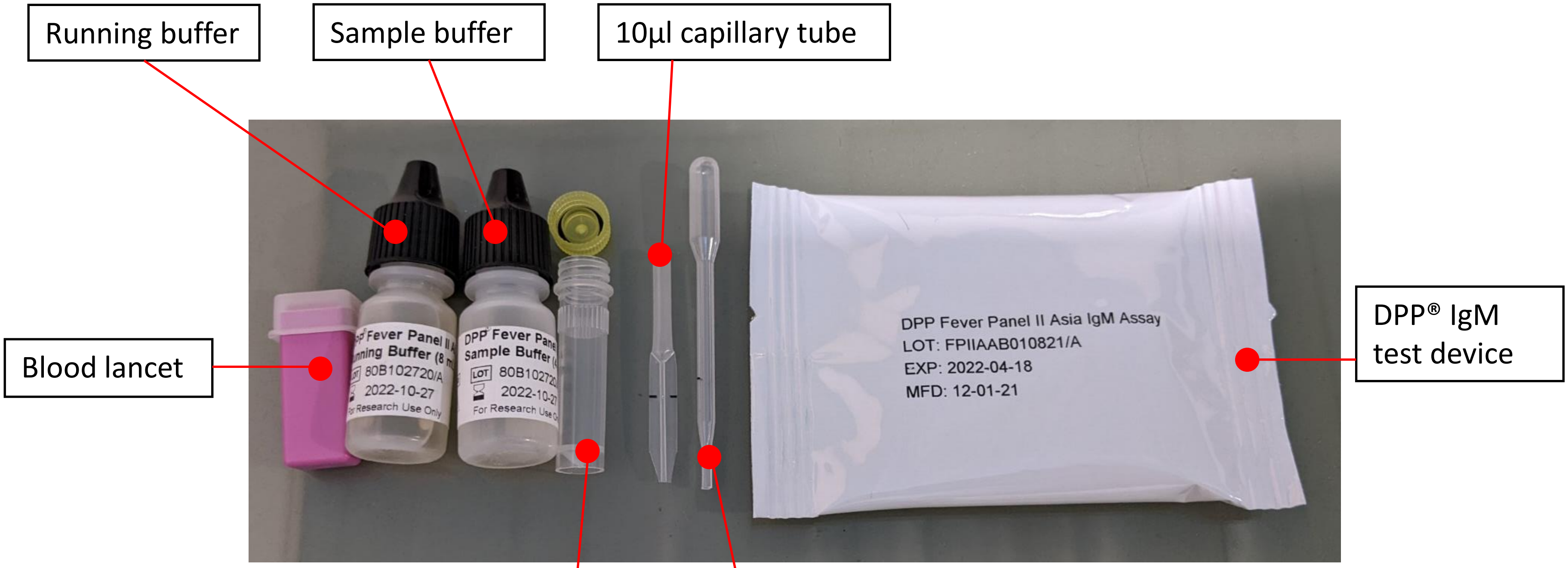

Not supplied in the box:
- Gloves
- Alcohol wipe
- Sharps disposal
- Timer

DPP® IgM system

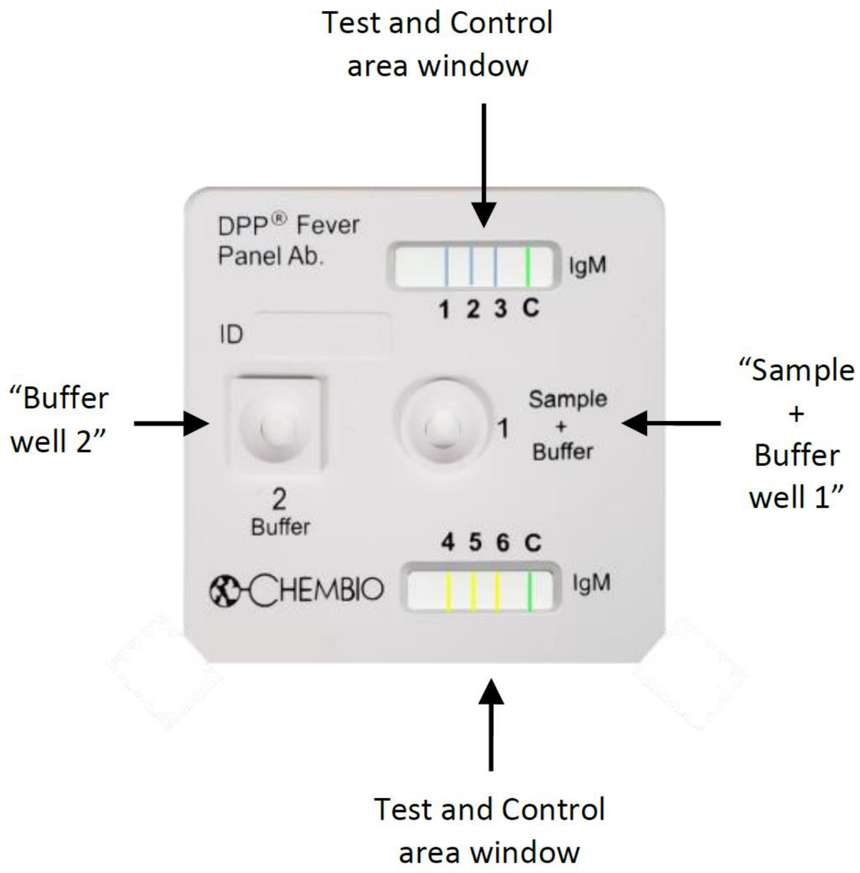

IgM Detection

1. Chikungunya
2. Zika
3. Leptospira
4. Orientia tsutsugamushi (scrub typhus)
5. Rickettsia typhi (murine typhus)
6. Dengue

DPP® IgM system: doing the test

1. Put 5 drops of the Sample Buffer into the sample vial.

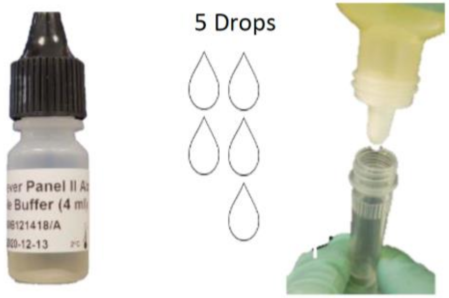

2. Collect the blood by touching it with the open end of the 10µl capillary tube. Hold it horizontally and let blood seep up to the black line.
   Do not squeeze the tube bulb.

Figure 1: Capillary Pipette

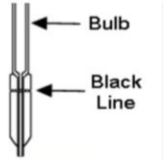

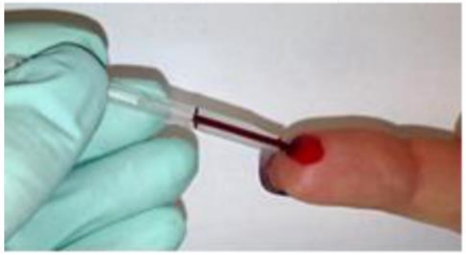

Figure 2: Capillary Pipette filled with Blood

DPP® IgM system: doing the test

3. Transfer the blood in the Microsafe® tube into the sample vial containing the Sample Buffer and squeeze the bulb to release the blood into the buffer. Discard the Microsafe® tube.

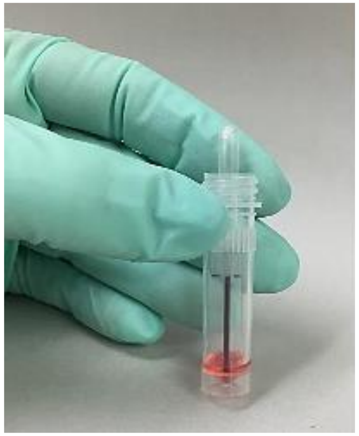

4. Using the 100µl transfer pipette, mix the blood and the buffer by pipetting up and down 6-8 times. Be careful not to introduce air bubbles into the solution.
5. Using the same transfer pipette, fill up to the black line.

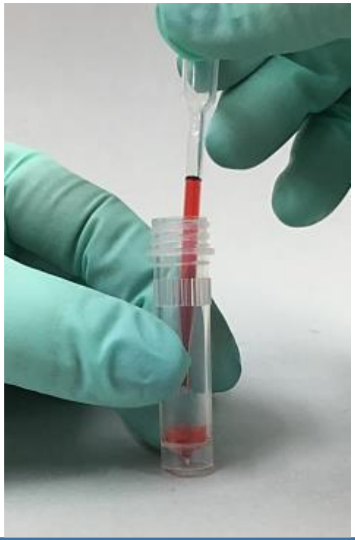

DPP® IgM system: doing the test

6. Release all the blood-buffer solution onto the “Sample+Buffer Well 1” by squeezing the tube bulb.

7. Start a timer immediately.

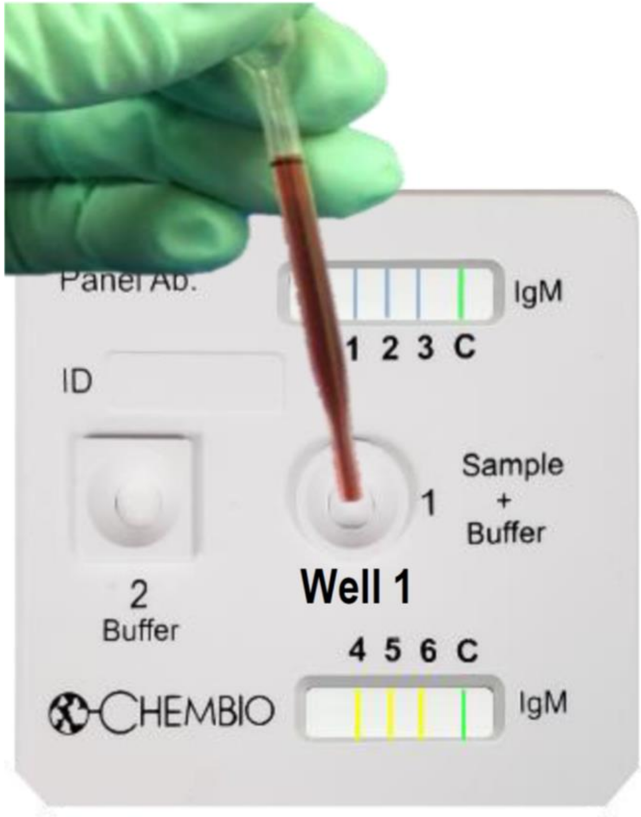

DPP® IgM system: doing the test

5. At **5 minutes**, add 12 drops the DPP® IgM Running Buffer to the “Buffer Well 2".

6. At **20-25 minutes**, read the results with the DPP® Micro Reader 2.
   Do not read after 25 minutes.

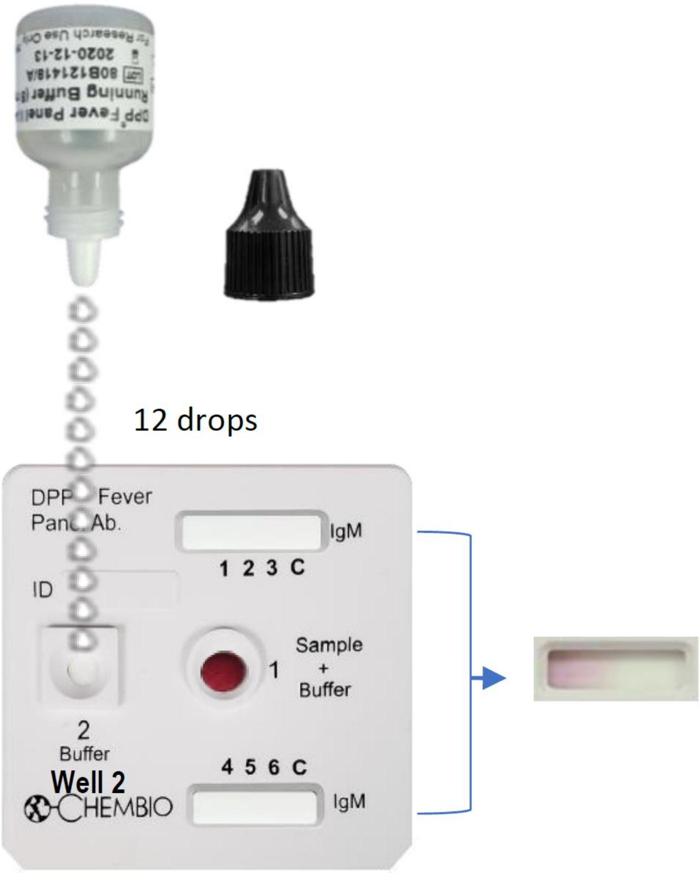

MORU Tropical Health Network
wellcome
UNIVERSITY OF OXFORD

Reading the results

All results must be read using the DPP® Micro Reader 2.
Do not read the DPP® Fever Panel result visually.

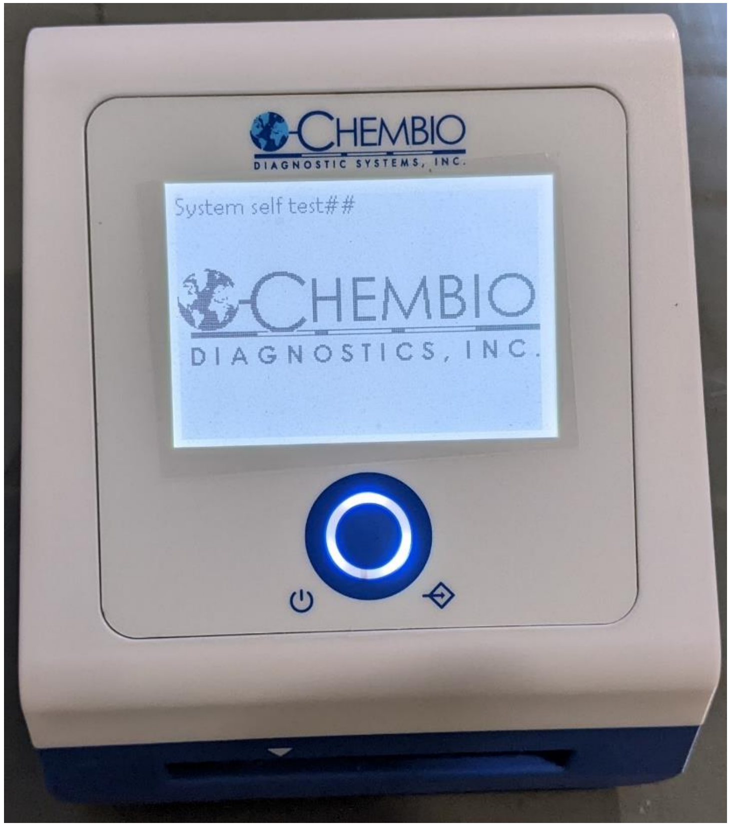

Reading the results

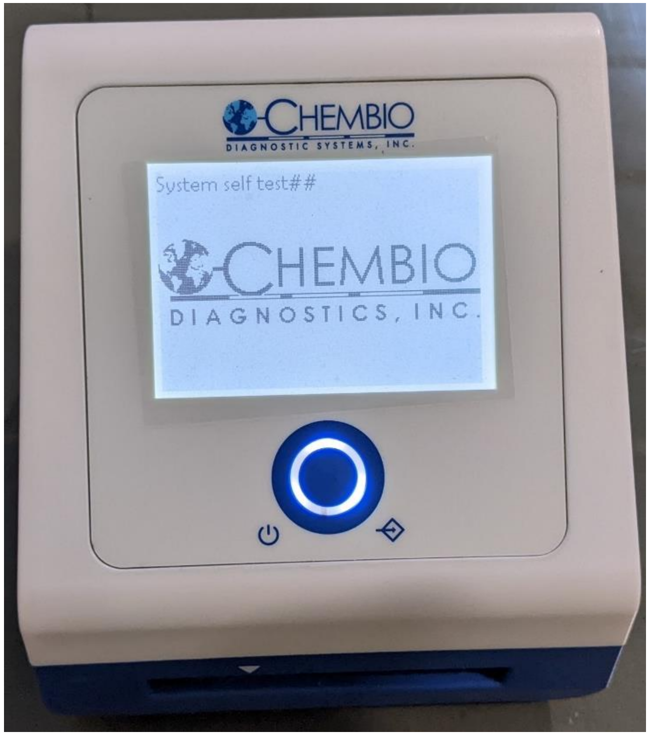

DPP® Micro Reader 2

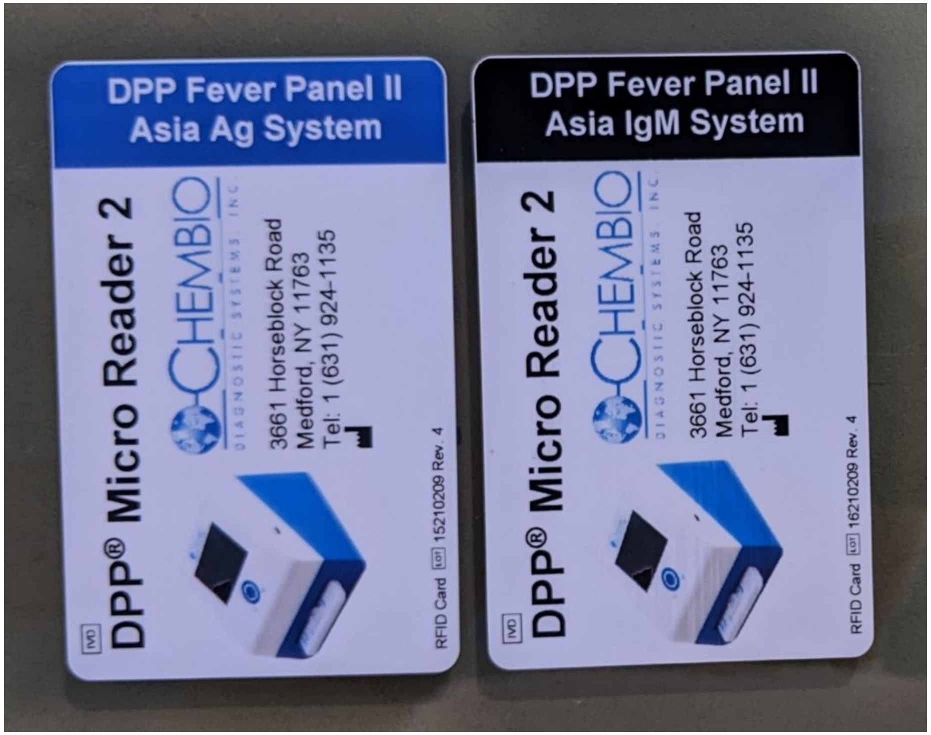

RFID cards
For the Antigen system (left, blue);
for the IgM system (right, black)

Reading the results

The Reader must be "programmed" using the appropriate RFID card before reading the test result.

- Use the Antigen (blue) RFID card before reading an Antigen test kit
- Use the IgM (black) RFID card before reading an IgM test kit

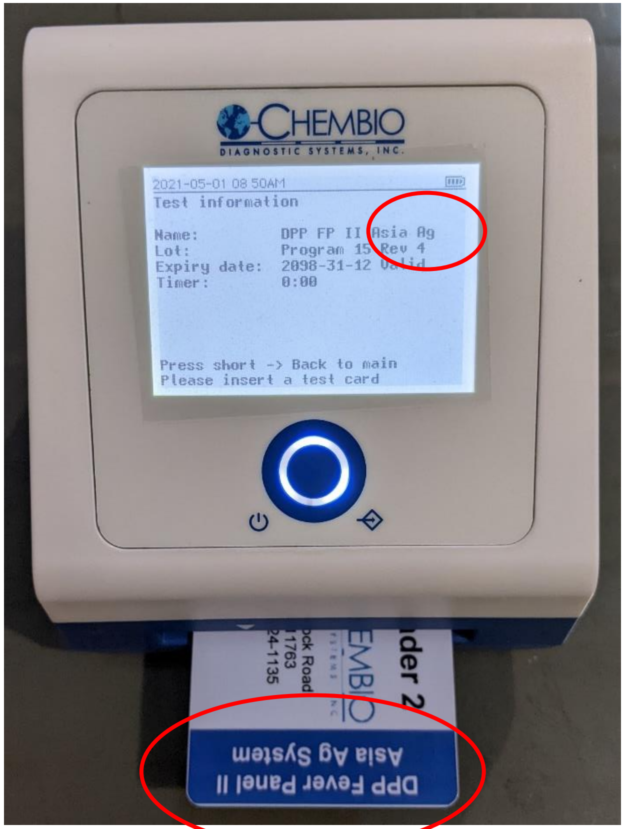

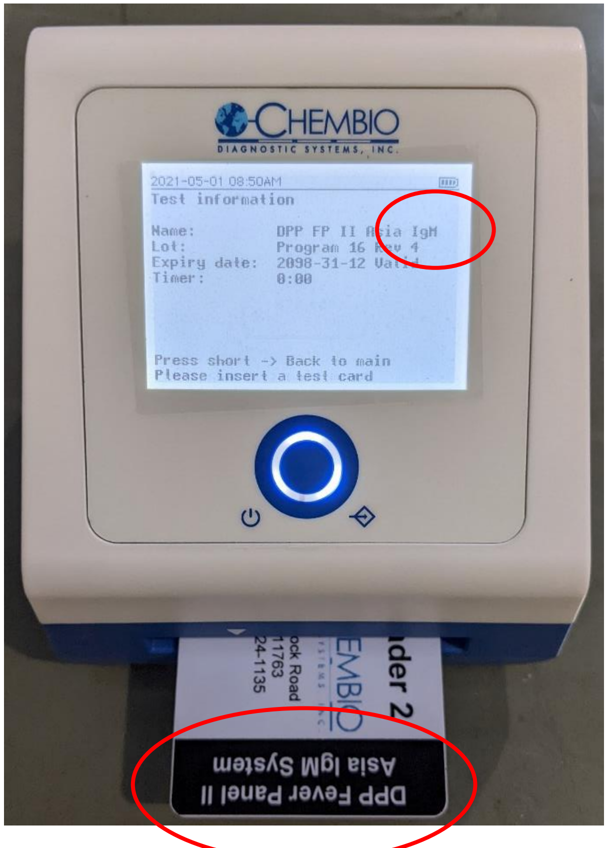

Reading the results

Results are numbers for each disease that must be compared to a reference table.

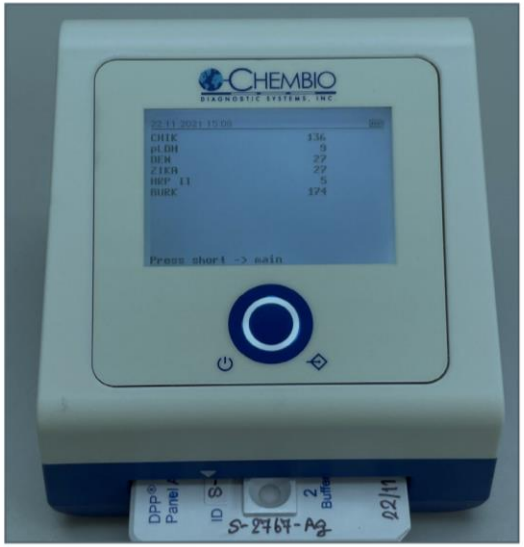

TEST RESULT

22/11/2021, 15:09

CHIK	136
pLDH	9
DEN	27
ZIKA	27
HRP II	5
BURK	174

Press short → main
